# Identification of Potential Therapeutic Targets Against Anthrax-Toxin-Induced Liver and Heart Damage

**DOI:** 10.3390/toxins17020054

**Published:** 2025-01-24

**Authors:** Lihong Wu, Yanping Chen, Yongyong Yan, Haiyan Wang, Cynthia D. Guy, John Carney, Carla L. Moreno, Anaisa Quintanilla-Arteaga, Fernando Monsivais, Zhichao Zheng, Mingtao Zeng

**Affiliations:** 1 Center of Emphasis in Infectious Diseases, Department of Molecular and Translational Medicine, Paul L. Foster School of Medicine, Texas Tech University Health Sciences Center El Paso, El Paso, TX 79905, USA; 2 Liver and GI Pathology Section, Department of Pathology, Duke University Medical Center, Durham, NC 27710, USA; 3 L. Frederick Francis Graduate School of Biomedical Sciences, Texas Tech University Health Sciences Center El Paso, El Paso, TX 79905, USA

**Keywords:** anthrax edema toxin, anthrax lethal toxin, *Bacillus anthracis*, cardiomyocytes, hepatocytes, microarray, organ damage, plasminogen activator inhibitor 1, small interfering RNA, therapeutic targets

## Abstract

Anthrax represents a disease resulting from infection by toxin-secreting bacteria, *Bacillus anthracis*. This research aimed to identify new therapeutic targets to combat anthrax. We performed assays to assess cell viability, apoptosis, glycogen consumption, and compound uptake and release in hepatocytes and cardiomyocytes responding to anthrax toxins. Microarray analysis was carried out to identify the genes potentially involved in toxin-induced toxicity. Knockdown experiments were performed to validate the contributions of the identified genes. Our study showed that anthrax edema toxin (EdTx) and lethal toxin (LeTx) induced lethal damage in mouse liver and heart, respectively. Microarray assays showed that 218 genes were potentially involved in EdTx-mediated toxicity, and 18 genes were potentially associated with LeTx-mediated toxicity. Among these genes, the knockdown of *Rgs1*, *Hcar2*, *Fosl2*, *Hcar2*, *Cxcl2*, and *Cxcl3* protected primary hepatocytes from EdTx-induced cytotoxicity. Plasminogen activator inhibitor 1 (PAI-1)-encoding *Serpine1* constituted the most significantly upregulated gene in response to LeTx treatment in mouse liver. PAI-1 knockout mouse models had a higher tolerance to LeTx compared with wild-type counterparts, suggesting that PAI-1 is essential for LeTx-induced toxicity and might represent a therapeutic target in LeTx-induced tissue damage. These results provide potential therapeutic targets for combating anthrax-toxin-induced liver and heart damage.

## 1. Introduction

Anthrax represents a disease resulting from infection by *Bacillus anthracis*, posing a serious threat to human life [1,2,3]. *B. anthracis*, or its spore, is a potential bioterrorism agent [4,5]. Anthrax foci can infect animals and humans with adequate quantities of virulent spores in the soil even after a long time [6]. Although some antibiotics have been used to treat patients with anthrax by directly killing *B. anthracis*, the outcomes remain poor, because anthrax toxins alter the functions of multiple organs and cause septic shock and death in the host [7]. Furthermore, the estimated mortalities of cutaneous, gastrointestinal, inhalational, and injectional anthrax are 1%, from 25 to 60%, 46%, and 33%, respectively, according to the outbreaks of *Bacillus anthracis* infection [8]. Therefore, it is urgently required to identify new therapeutic targets to combat anthrax-toxin-induced organ damage.

*B. anthracis* gains virulence through a three-component protein exotoxin comprising protective antigen (PA), edema factor (EF), and lethal factor (LF) [7,9]. EF and LF are individually non-toxic, but are toxic in combination with PA to form two A/B toxins, edema toxin (EdTx, EF + PA) and lethal toxin (LeTx, LF + PA), causing different pathogenic responses in cultured cells and animals. In these two toxins, the A components, EF and LF, have enzymatic activities. EF in EdTx constitutes a calmodulin-dependent adenylate cyclase elevating intracellular cAMP levels, leading to subcutaneous edema and fluid accumulation in organs [7,10]. LF in LeTx represents a zinc-dependent metalloprotease that specifically cleaves the N-terminal end of many mitogen-activated protein kinase kinases such as MAPKK or MEK [11,12,13,14]. These disrupted pathways are critical for cell proliferation, cell cycle regulation, and immune function. The B component, PA, binds to anthrax toxin receptors on cells, including tumor endothelium marker 8 (TEM8) and capillary morphogenesis protein 2 (CMG2) [15]. Therefore, anthrax toxins exert a wide range of toxic effects by increasing intracellular cAMP and/or the cleavage of MEKs. Although the signaling pathways involved in anthrax-toxin-induced organ damage have been extensively investigated [16,17,18], its underlying mechanisms and downstream targets are poorly understood.

Liu et al. reported that anthrax toxins selectively induce damage in distinct cell types. EdTx mainly targets the liver and induces unique liver edema that does not occur in other organs. Meanwhile, LeTx targets cardiomyocytes and vascular smooth muscle cells, leading to LeTx-induced mortality [19]. In this study, we focused on the effects of anthrax toxins on the cellular functions and signaling pathways in mouse liver and heart, using cell culture and animal experiments. Potential target genes involved in anthrax-toxin-induced cell and organ damage were identified using bioinformatics methods. Small interfering RNA (siRNA)-mediated gene silencing and a knockout mouse model were utilized to investigate potential protective strategies against anthrax toxins.

## 2. Results

### 2.1. EdTx Rapidly Impairs Liver Function in A/J Mice

**Figure 1:**
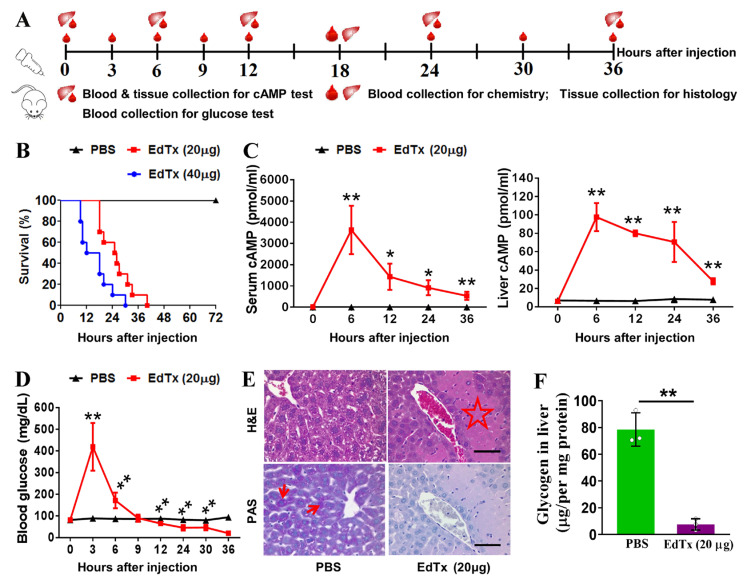
EdTx rapidly degrades liver function in A/J mice. A/J mice were intravenously injected with 20 µg EdTx (20 µg EF plus 40 µg PA) or 40 µg EdTx in 0.2 mL PBS, while mice in the control group were injected with PBS only. (**A**) Animal experiment flow chart. (**B**) Survival curves (n = 10). (**C**) The levels of cAMP in serum (left) and liver tissue (right) at different time points after EdTx (20 µg) challenge (n = 15). (**D**) Blood glucose level at different time points after EdTx (20 µg) challenge. (**E**) H&E (top) and PAS (bottom) staining of liver tissues at 18 h after EdTx (20 µg) challenge. Scale bar, 30 µm. Red arrows indicate glycogen in the liver. The red star indicates hepatocellular necrosis in the liver. n = 3. (**F**) Glycogen concentration in liver at 18 h after EdTx (20 µg) challenge. * *p* < 0.05, ** *p* < 0.01 vs. PBS control group; n = 3.

The A/J strain of mice is widely used in anthrax vaccine research due to its immune response to recombinant protective antigens [20]. To examine the impact of EdTx on liver function in vivo, A/J mice were administered with 20 or 40 μg EdTx, and blood and tissue samples were collected at different time points (Figure 1A). We found that the A/J mice were extremely vulnerable to EdTx and showed symptoms starting 30–60 min after injection. Mice receiving 20 or 40 μg EdTx started to die within 40 h and 30 h after injection, respectively (Figure 1B). Considering the relatively long survival time in response to 20 μg EdTx versus 40 μg EdTx, we used 20 μg EdTx in the following experiments.

To evaluate the liver function of A/J mice challenged with EdTx, we monitored their circulating and hepatic cAMP levels. As shown in Figure 1C, compared with those in PBS-injected mice, the cAMP levels in the EdTx-injected mice rapidly increased after injection, peaked at 6 h (3636.8 pmol/mL vs. 3.2 pmol/mL in serum, *p* < 0.01; 780.8 pmol/mL vs. 52.7 pmol/mL in the liver, *p* < 0.01), and gradually decreased after that, but still remained significantly higher than those in the control mice until death. Similarly, the level of blood glucose rapidly increased after EdTx injection, peaked at 3 h (419.4 mg/dL vs. 89.4 mg/dL in control, *p* < 0.01), quickly declined to control values (85.7 mg/dL) at nearly 9 h, and further declined until death (Figure 1D).

To further evaluate the effects of EdTx on liver function, we analyzed blood chemistry at 18 h after the EdTx challenge. As shown in Supplementary Table S1, the blood levels of liver function indicators, including aspartate aminotransferase (AST), alanine aminotransferase (ALT), alkaline phosphatase (ALP), and creatine kinase (CK), were significantly higher in the EdTx-challenged mice than those in the control mice. Albumin (ALB), globulin (GLB), and total protein (TP), which are mainly synthesized in the liver, were markedly lower in the EdTx-challenged mice than those in the control mice. Moreover, the renal function indexes, e.g., blood urea nitrogen (BUN), creatinine (CREA), phosphorous, and potassium, were significantly elevated, whereas blood calcium was notably reduced in the EdTx-treated mice. Taken together, these results suggest that EdTx challenge leads to the acute deterioration of liver function in A/J mice.

We further evaluated whether EdTx induces hepatic damage in A/J mice by performing H&E staining in the liver tissue collected from the mice at 18 h after the challenge with 20 μg EdTx. As shown in Figure 1E (upper panel), the livers of the EdTx-treated mice demonstrated characteristic signs of hepatocellular necrosis, such as “geographic shape”, eosinophilia, islands around the central hepatic veins, and a thin rim of surviving hepatocytes close to the vein wall. Many of these lesions were asymmetrically distributed around the circumference of the vein. PAS staining showed dramatically decreased fuchsia staining within the livers of the EdTx-treated mice compared with those of the control mice (Figure 1E, lower panel), suggesting a decrease in liver glycogen storage in response to the EdTx challenge. This was further confirmed by glycogen assay (Figure 1F). Collectively, these results suggest that EdTx induces acute hepatic damage in vivo, leading to a deterioration in liver function in A/J mice.

### 2.2. Anthrax Toxin Receptors Are Expressed in Mouse Liver and Primary Hepatocytes

**Figure 2:**
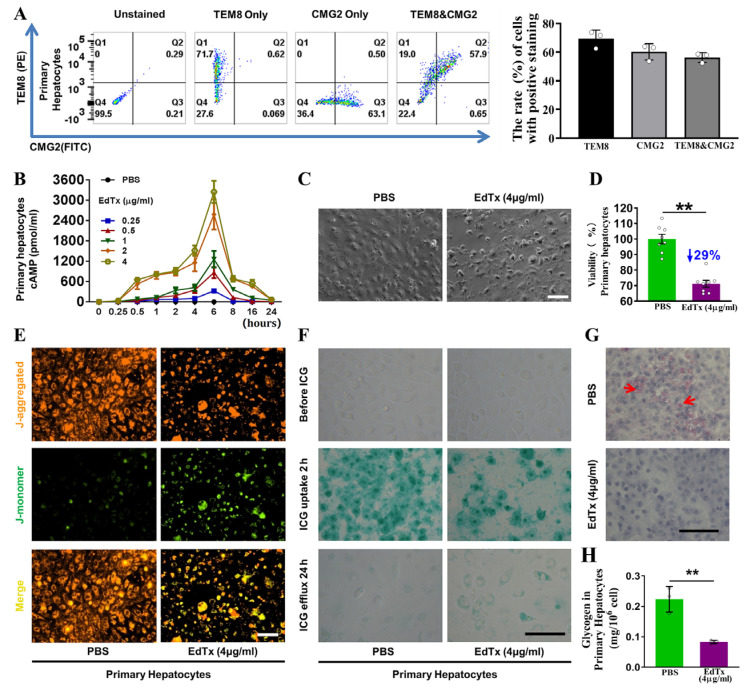
EdTx inhibited cell viability, promoted cell apoptosis, and induced cytotoxicity in primary hepatocytes. (**A**) Flow cytometry analysis was performed to measure the expression of the anthrax toxin receptors TEM8 and CMG2 in primary hepatocytes. Representative plots (left panel) are shown for cell samples that were unstained, stained for TEM8 alone, for CMG2 alone, or dual-stained for TEM8 and CMG2. The results were quantified (right panel). (**B**) Primary hepatocytes were treated with 0.25, 0.5, 1, 2, or 4 µg/mL EdTx and then lysed with 0.5 mL of 0.1 M HCl at 0, 0.25, 0.5, 1, 2, 4, 6, 8, 16, or 24 h, with PBS used as a negative control. Cell lysates were diluted 8-fold for testing. The level of intracellular cAMP was measured using a commercial ELISA kit. Representative results from three independent experiments are shown. Data are expressed as mean ± standard deviation. ** *p* < 0.01 vs. PBS. (**C**) Primary hepatocytes were treated with PBS or EdTx (4 µg/mL) for 6 h, and cells were visualized using phase-contrast microscopy. Scale bar, 100 µm. (**D**) An MTT assay was performed after primary hepatocytes were treated with PBS or EdTx (4 µg/mL) for 6 h. Representative results from three independent experiments are shown. Data are normalized to the cell viability of PBS-treated control cells and expressed as mean ± standard deviation. ** *p* < 0.01 vs. control; n = 8. (**E**) Mitochondrial membrane potential analysis using 5,5′,6,6′-tetrachloro-1,1′,3,3′-tetraethyl benzimidaloyl carbocyanine iodide (JC-1) mitochondrial membrane dye. Cells were treated with PBS or EdTx (4 µg/mL) for 6 h and then incubated with 10 µg/mL JC-1 in a CO_2_ incubator at 37 °C for 30 min. Normal cells appear red with the J-aggregated stain, and apoptotic cells appear green with the J-monomer stain. Scale bar, 100 µm. (**F**) Indocyanine green (ICG) uptake-and-release assay. Cells were treated with PBS or EdTx (4 µg/mL) for 6 h and incubated with ICG in a CO_2_ incubator at 37 °C for 2 h. Medium was refreshed after 24 h of incubation. Cells with a green-stained nucleus are ICG-positive hepatocytes. Scale bar, 100 µm. (**G**) PAS staining. Cells were treated with PBS or EdTx (4 µg/mL) for 6 h and stained for glycogen within the cells using the Sigma-Aldrich PAS kit. Scale bar, 100 µm. Red arrows indicate glycogen in the cells. (**H**) Quantification of (**G**). Data are expressed as mean ± standard deviation. ** *p* < 0.01 vs. control; n = 3.

Since anthrax toxin receptors are required for the entry of anthrax toxin into cells, we assessed the expression levels of the anthrax toxin receptor-encoding genes *Tem8* and *Cmg2* in primary hepatocytes and mouse liver. As shown in Supplementary Figure S1, transcripts of both *Tem8* and *Cmg2* were detectable in the primary hepatocytes and liver tissue of the A/J mice. Consistently, the results of flow cytometry showed the presence of TEM8 and CMG2 proteins on the surface of primary hepatocytes (Figure 2A). These results indicate the presence of anthrax toxin receptors on hepatocytes, which provides the necessary mechanism for anthrax toxin delivery into hepatocytes.

### 2.3. EdTx Inhibits Cell Viability, Promotes Cell Apoptosis, and Induces Cytotoxicity in Primary Hepatocytes

To examine the effects of EdTx on primary hepatocytes, we determined the intracellular cAMP levels at different time points within 24 h after treatment with 0.25, 0.5, 1, 2, or 4 µg/mL of EdTx. PBS was used as a negative control. As shown in Figure 2B, compared with that observed in control cells, the level of cAMP was dramatically increased in response to EdTx, dose-dependently, peaking at 6 h after EdTx treatment and rapidly decreasing to baseline at 24 h after EdTx treatment, regardless of the dose. An abnormal cell morphology, granulation of the cytoplasm, contraction, disconnection, and cellular debris were observed at 6 h after treatment with 4 µg/mL of EdTx (Figure 2C). Consistently, the MTT assay revealed that cell viability in primary hepatocytes administered with 4 µg/mL of EdTx for 6 h was remarkably reduced by 29% compared with those treated with PBS (*p* < 0.01; Figure 2D). These results indicate that EdTx quickly induces cytotoxicity in hepatocytes.

To assess whether this reduced primary hepatocyte viability was related to enhanced cell apoptosis, the mitochondrial membrane potential assay was carried out. As shown in Figure 2E, EdTx reduced red fluorescence (J-aggregates) and increased green fluorescence (J-monomers) due to the selective entry of J-monomers into the mitochondria, indicating a decrease in mitochondrial membrane potential in response to EdTx treatment (Figure 2E). These results suggest that EdTx induces cell apoptosis in primary hepatocytes, resulting in the suppression of cell survival.

Hepatocytes are critical for the metabolism of numerous compounds. To further examine the effect of EdTx on liver function, we performed an indocyanine green (ICG) uptake-and-release assay. As shown in the middle panel of Figure 2F, at 2 h after treatment with PBS or 4 µg/mL of EdTx, approximately 84.00% of the PBS-treated cells absorbed ICG and exhibited a green-stained nucleus, whereas only 38.94% of the EdTx-treated cells had the ability to take up ICG (*p* < 0.01). At 24 h after treatment, the amounts of ICG-positive cells were dramatically reduced in both groups. However, the green color was more intense in the EdTx-treated cells compared with the PBS-treated cells (Figure 2F, lower panel), suggesting an impaired ability of the EdTx-treated hepatocytes to take up and release ICG. We further examined the glycogen storage capacity of the primary hepatocytes using PAS staining. As shown in Figure 2G,H, the EdTx-treated cells exhibited less storage of glycogen than the PBS-treated cells (*p* < 0.01). Jointly, these findings suggest that EdTx rapidly induces cytotoxicity in primary hepatocytes, leading to an impaired uptake, release, and storage of compounds.

### 2.4. Identification of EdTx Cytotoxicity-Related Genes

**Figure 3:**
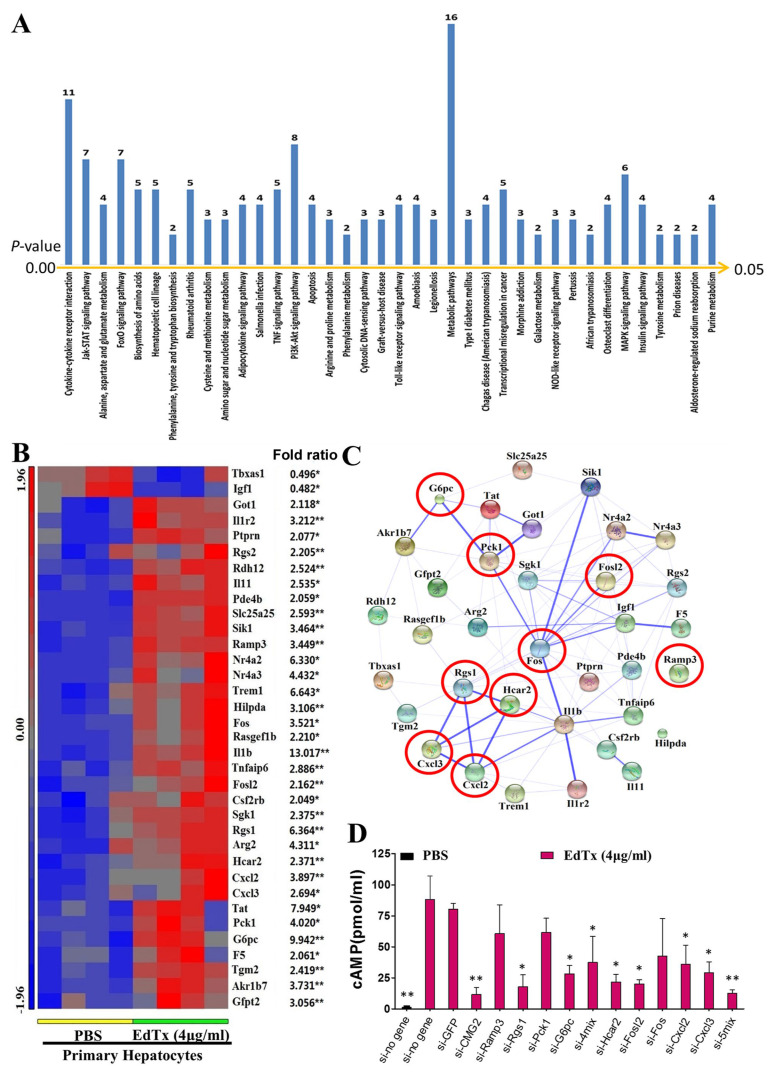
Identification of EdTx-induced, cytotoxicity-related genes. Total RNA was isolated from primary hepatocytes treated with PBS or EdTx (4 μg/mL) for 6 h, and the samples were subjected to microarray analysis using the GeneChips Mouse Transcriptome Assay 1.0. Some genes (218) were found to have significant expression changes in primary hepatocytes exposed to EdTx treatment compared with PBS-treated cells. The Partek Genomics Suite was used to analyze the signaling pathways associated with these differentially expressed genes. (**A**) The pathways with enrichment score > 2 and *p*-value < 0.05 are shown. The numbers at the top of each column show the number of genes that have expression changes in each pathway. (**B**) The 35 significantly changed genes and sequencing expression fold changes. * *p* < 0.05, ** *p* < 0.01 vs. PBS. (**C**) The results of a protein–protein network analysis among the 35 genes are shown. Nine genes that are involved in glycogen metabolism, cAMP production, and cell apoptosis are marked with red circles and were further investigated in the following experiments. (**D**) These potential EdTx-induced cytotoxicity-related genes were knocked down individually or in combination in primary hepatocytes using the corresponding siRNAs. si-CMG2 was used as a positive control, and si-GFP and si-(no gene) were used as negative controls. Primary hepatocytes deficient in these genes were treated with PBS or EdTx (4 μg/mL) for 6 h. The intracellular concentration of cAMP was determined using ELISA. * *p* < 0.05, ** *p* < 0.01 vs. si-GFP (n = 3). 4mix, Ramp3 + Rgs1 + Pck1 + G6pc; 5mix, Hcar2 + Fosl2 + Fos + Cxcl2 + Cxcl3.

To investigate the mechanism underlying EdTx-induced cytotoxicity in hepatocytes, we conducted a microarray assay to identify differentially expressed genes in response to EdTx treatment. We identified 218 differentially expressed genes (log ratio > 2 or log-ratio < 0.5, *p* < 0.05) and 38 significantly changed pathways (enrichment score > 2, *p* < 0.05; Figure 3A) in EdTx-treated primary hepatocytes compared with PBS-treated cells (Supplementary Table S2). The accession number of the microarray data in the Gene Expression Omnibus (GEO) is GSE115844.

To verify the microarray results, we performed qPCR to detect 70 genes selected from the 38 signaling pathways using the same RNA samples as those in the microarray assay. Of note, the expression changes of 35 genes were confirmed (*p* < 0.05 vs. control) in both primary hepatocytes and liver tissue (Supplementary Table S2). The microarray results of the 35 genes are shown in Figure 3B. To further understand the associations among the 35 genes, a protein–protein network analysis was conducted with STRING 10. Strong associations were detected among *Akr1b7*, *G6pc*, *Pck1*, *Got, Fos*, *Fosl2*, *Sik1*, *Nr4a2*, *Nr4a3*, *Igf1*, *F5*, *I11b*, *Tnfaip6*, *I11r2*, *Rgs1*, *Hcar2*, *Cxcl2*, and *Cxcl3* (Figure 3C, Supplementary Figure S1). Based on our preliminary data, we selected nine genes, including *G6pc*, *Pck1*, *Fosl2*, *Ramp3*, *Fos*, *Rgs1*, *Hcar2*, *Cxcl2*, and *Cxcl3*, that are involved in glycogen metabolism, cAMP production, and cell apoptosis, to investigate whether they were related to EdTx-induced cytotoxicity in the primary hepatocytes. The efficiency of the siRNA-mediated knockdown of each gene is shown in Supplementary Table S3. Sequence information for the used siRNAs is provided in Supplementary Table S4. An EdTx-induced increase in the intracellular cAMP level is considered to be an indicator of EdTx cytotoxicity [21]. Using *Cmg2* as a positive control, we found that the intracellular cAMP level was dramatically increased in si-control- or si-GFP-transfected cells at 1 h after EdTx treatment in comparison with PBS-treated cells (Figure 3D). Interestingly, in EdTx-treated cells, the knockdown of *Cmg2*, *Rgs1*, *Hcar2*, *Fosl2*, *Cxcl2*, *xcl3*, *Ramp3* + *Rgs1* + *Pck1* + *G6pc*, or *Hcar2* + *Fosl2* + *Fos* + *Cxcl2* + *Cxcl3* significantly reversed the effect of EdTx on the cAMP level. These data suggest that these genes are essential for EdTx-induced cAMP production, and that the knockdown of these genes, individually or in combination, may protect hepatocytes from EdTx-induced cytotoxicity.

### 2.5. LeTx Induces Liver Toxicity In Vivo

To assess LeTx toxicity in vivo, we injected C57BL/6J mice (n = 10), which is the background strain of the PAI-1^−/−^ mice used in the following study, with PBS or different doses of LeTx (8.75, 12.5, 18.75, 25, or 50 μg/mouse). As shown in Figure S1A, all mice died within 2–8 days, except those treated with PBS or the lowest dose of LeTx (8.75 μg/mouse), suggesting that LeTx is toxic and lethal to mice in a dose-dependent manner (Figure S2A). Because LeTx cleaves the N-terminus of MEK2, we detected MEK2 cleavage with an antibody targeting this N-terminus [15]. As shown in Figure S2B, in the heart and liver samples of mice that were administered with LeTx for 24 h, the protein expression of cleaved MEK2 was absent in both tissue samples compared with those treated with PBS, suggesting that LeTx is biologically functional in vivo.

To further evaluate the effects of LeTx on liver function, we conducted a blood chemistry analysis at 24 h after LeTx (50 μg/mouse) challenge. As shown in Supplementary Table S1, starkly higher amounts of the liver enzymes AST, ALT, and CK were found in the LeTx-treated mice compared with PBS-treated animals (AST, 398.00 ± 162.01 vs. 66.75 ± 8.62; ALT, 172.50 ± 40.12 vs. 42.75 ± 8.88; CK, 1960.50 ± 980.46 vs. 616.50 ± 109.42; all *p* < 0.05). By contrast, the levels of ALB, GLB, and TP, which are mainly synthesized in the liver, were remarkably lower in the LeTx-challenged mice in comparison with control animals. These results suggest that LeTx impairs liver function in C57BL/6J mice.

### 2.6. Anthrax Toxin Receptors Are Expressed in Mouse Heart and Primary Cardiomyocytes

**Figure 4:**
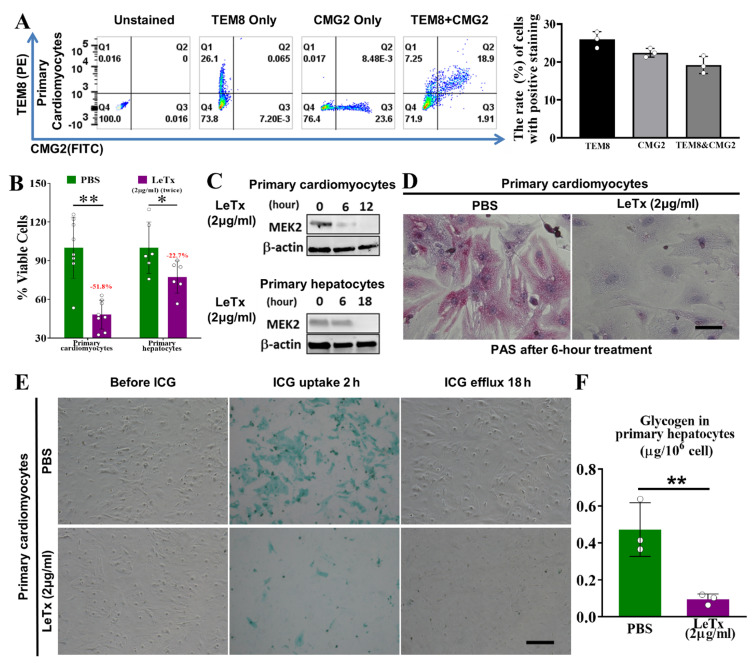
LeTx suppresses cell survival and induces cytotoxicity in vitro. (**A**) Flow cytometry analysis was performed to measure the expression of the anthrax toxin receptors TEM8 and CMG2 in primary cardiomyocytes. Representative plots (left panel) are shown for cell samples that were unstained, stained for TEM8 alone, stained for CMG2 alone, or dual-stained for TEM8 and CMG2, and the results were quantified (right panel). Experiments were independently repeated at least three times. Data are expressed as mean ± standard deviation (SD). (**B**) Two doses of LeTx (2 μg/mL) were administered with an 18 h interval between doses. An MTT assay was performed at 18 h after the second dose of LeTx to examine cell viability. The data are expressed as mean ± SD. * *p* < 0.05, ** *p* < 0.01 vs. PBS-treated control cells; n = 8. (**C**) Western blot assay for cleaved MEK2 and β-actin expression in primary cardiomyocytes and hepatocytes at 0, 6, 12, or 18 h after LeTx (2 μg/mL) treatment. (**D**) PAS staining assy. Primary cardiomyocytes were treated with PBS or LeTx (2 µg/mL) for 6 h and stained for glycogen within the cells using the Sigma-Aldrich PAS kit. Scale bar, 100 µm. (**E**) Indocyanine green (ICG) uptake-and-release assay. Primary cardiomyocytes were treated with PBS or LeTx (2 µg/mL) for 6 h and incubated with ICG in a CO_2_ incubator at 37 °C for 2 h. Medium was refreshed after 18 h of incubation. Cells with green-stained nuclei are ICG-positive cardiomyocytes. Scale bar, 100 µm. (**F**) Quantification of (**D**). Data are expressed as mean ± SD. ** *p* < 0.01 vs. control; n = 3.

To confirm the existence of anthrax toxin receptors on cardiomyocytes, we performed qRT-PCR and flow cytometry analyses using primary cardiomyocytes and heart tissue samples from C57BL/6J mice. As shown in Supplementary Figure S3 both *Tem8* and *Cmg2* transcripts were detectable in the primary cardiomyocytes and heart tissue of C57BL/6J mice. Consistently, the results of flow cytometry showed the presence of TEM8 and CMG2 proteins on the primary cardiomyocytes (Figure 4A). These results suggest the presence of anthrax toxin receptors on the cell surface of cardiomyocytes, which provide the necessary components for anthrax toxin delivery to cardiomyocytes.

### 2.7. LeTx Suppresses Cell Viability and Induces Cytotoxicity in Primary Hepatocytes and Primary Cardiomyocytes In Vitro

We next explored whether LeTx is toxic to primary hepatocytes and primary cardiomyocytes in vitro using the MTT assay. We failed to obtain any useful data by treating the cells with a single dose of LeTx (2 μg/mL) for 12 h or 18 h (Supplementary Figure S4). Thus, the cells were treated with two doses of LeTx (2 μg/mL), with an 18 h interval. The results showed that, at 18 h after the second dose, when compared with those of PBS-treated cells, the cell viabilities of LeTx-treated primary cardiomyocytes and hepatocytes were significantly inhibited by 51.8% (*p* < 0.01) and 22.7% (*p* < 0.05), respectively (Figure 4B).

To assess the impacts of LeTx on cardiomyocyte and hepatocyte function involving the metabolism of compounds, we performed an MEK2 cleavage assay, ICG uptake-and-release assay, and PAS staining. As shown in Figure 4C, MEK2 was completely cleaved within 12 h and 18 h after LeTx treatment (2 μg/mL) in primary cardiomyocytes and hepatocytes, respectively. PAS staining revealed that, after treatment with LeTx (2 μg/mL) for 6 h, the glycogen storage (fuchsia staining) in primary cardiomyocytes was dramatically decreased by nearly 90% compared with that in PBS-treated cells (Figure 4D,F). The results of the ICG uptake-and-release assay demonstrated that primary cardiomyocytes treated with 4 µg/mL of LeTx had less ability to take up ICG than PBS-treated cells (Figure 4E). Collectively, these results indicate that a high dose of LeTx induces cytotoxicity in cardiomyocytes, leading to a suppressed cell viability and impaired cell functions.

### 2.8. Identification of LeTx Cytotoxicity-Related Genes

**Figure 5:**
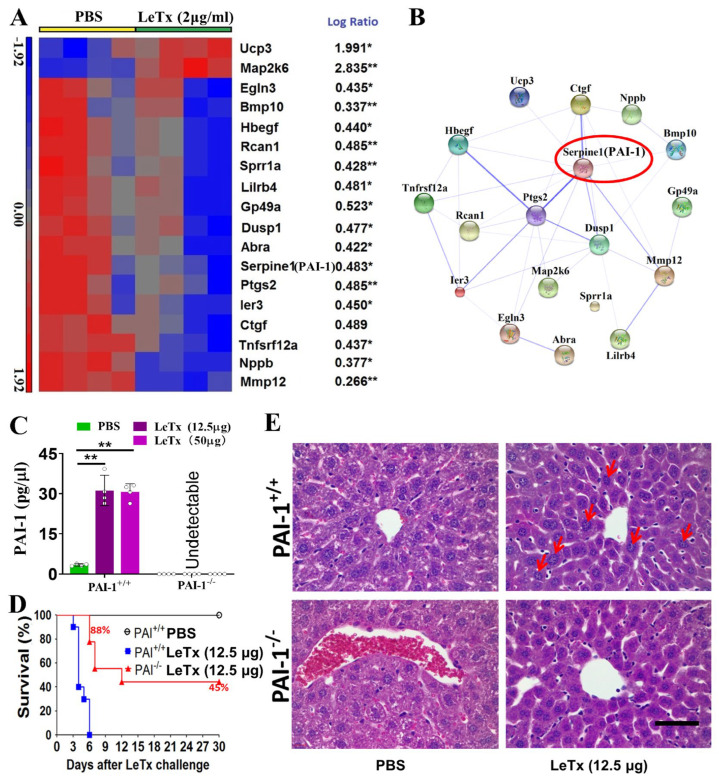
Identification of LeTx-induced, cytotoxicity-related genes. (**A**) Heat map of 18 genes that have significant expression changes (with log-ratio > 1.8 or log-ratio < 0.56) in LeTx-treated primary cardiomyocytes. * *p* < 0.05, ** *p* < 0.01 vs. PBS. (**B**) The results of a protein–protein network analysis among these genes using STRING 10 software. Thick lines indicate strong associations. Serpine1 (encoding PAI-1), which is at the center of the network and marked with a red circle, was selected for knockout in subsequent experiments. (**C**) The serum levels of PAI-1 in WT Balb/c mice, WT C57BL/6J mice, and PAI-1 knockout (PAI-1^−/−^) C57BL/6J mice treated with 0, 12.5, or 50 µg LeTx were determined by ELISA. ** *p* < 0.01 vs. control. (**D**) Survival curve of WT and PAI-1^−/−^ C57BL/6J mice treated with PBS or 12.5 µg of LeTx. (**E**) H&E staining of liver sections from WT and PAI-1^−/−^ C57BL/6J mice treated with PBS or 12.5 μg LeTx. Scale bar, 30 µm. Red arrows indicate anisonucleosis in the liver. Scale bar, 100 µm.

To identify the genes that are potentially associated with the toxicity of LeTx in primary cardiomyocytes, we conducted a microarray assay using RNA samples from primary cardiomyocytes exposed to PBS or 2 μg/mL of LeTx for 18 h. The accession number of the microarray data in GEO is GSE116755. As shown in Figure 5A, we identified 18 differentially expressed genes (*Abra*, *Bmp10*, *Ctgf*, *Dusp1*, *Egln3*, *Gp49a*, *Hbegf*, *Ier3*, *Lilrb4*, *Map2k6*, *Mmp12*, *Nppb*, *Ptgs2*, *Rcan1*, *Serpine1*, *Sprr1a*, *Tnfrsf12a*, and U*cp3*; log-ratio > 1.8 or log-ratio < 0.56, *p* < 0.05) in LeTx-treated cells in comparison with PBS-treated cells. The protein–protein network analysis demonstrated that the 18 genes were associated with the MAPK, HIF-1, and TNF signaling pathways (Figure 5B). We next performed qRT-PCR to verify the microarray results using the same RNA samples. All qPCR primers are described in Supplementary Table S5. The results are shown in Supplementary Table S6 and were consistent with the microarray analysis.

Moreover, we detected the mRNA expression of the 18 genes in LeTx-treated mouse hearts, primary hepatocytes, and mouse livers. As shown in Supplementary Table S6, *Map2k6* was the only gene that LeTx significantly upregulated at the transcriptional level in all cells and tissue samples. On the other hand, the mRNA expressions of *Gp49a*, *Hbegf*, *Lilrb4*, and *Tnfrsf12a* were significantly downregulated by LeTx in all cells and tissue samples. Of note, the mRNA levels of *Dusp1*, *Ier3*, and PAI-1-encoding *Serpine1* were significantly lower in LeTx-treated primary cardiomyocytes than those in PBS-treated cells, but notably higher in LeTx-treated mouse livers compared with those in the control group. Importantly, PAI-1-encoding *Serpine1* was the most significantly upregulated gene (377-fold) in response to LeTx treatment in mouse liver compared with the control group (Supplementary Table S6), which prompted us further to investigate PAI-1’s role in LeTx-induced liver toxicity.

### 2.9. PAI-1^−/−^ Mice Are More Tolerant to LeTx than WT Mice

In Balb/c mice, consistent with the dramatic increase in the Serpine1 mRNA level in the livers of LeTx-treated mice, the serum level of PAI-1 was significantly increased in response to LeTx treatment (12.5 µg LeTx: 28.25 ± 5.50 pg/µL vs. 3.14 ± 0.16 pg/µL; 50 µg LeTx: 43.87 ± 9.39 pg/µL vs. 3.14 ± 0.16 pg/µL; all *p* < 0.01; Figure 5C). Because the PAI-1^−/−^ mice were from the C57BL/6J strain, we determined the serum level of PAI-1 in WT and PAI-1^−/−^ C57BL/6J mice. We found that serum PAI-1 levels were also markedly increased in response to LeTx treatment (Figure 5C). Both the WT and PAI-1^−/−^ groups died within 1 week after the challenge with 50 µg LeTx. However, only the WT C57BL/6J mice died within 6 days after the challenge with 12.5 µg LeTx, whereas 88% of the PAI-1^−/−^ C57BL/6J mice survived (*p* < 0.01; Figure 5D), suggesting that PAI-1 plays an essential role in LeTx-induced toxicity in the mouse model. H&E staining further revealed that, after 12.5 µg LeTx treatment, the livers of WT mice exhibited morphological abnormalities, such as anisonucleosis and centrilobular congestion, compared with the livers of PAI-1^−/−^ mice (Figure 5E), suggesting that PAI-1^−/−^ mice are more tolerant to 12.5 µg LeTx than WT mice.

## 3. Discussion

Here, the effects of EdTx and LeTx on mouse liver and heart functions were examined, and we identified differentially expressed genes that are associated with the toxicity of EdTx and LeTx, providing potential therapeutic targets for anthrax treatment.

Previous studies suggested that the effect of LeTx on cardiac function remains controversial. Lawrence et al. demonstrated that LeTx administration results in a reduced heart rate, decreased mean arterial pressure, and myodegeneration/necrosis and cardiac inflammation using the rabbit model [22]. On the other hand, Li et al. showed that EdTx, but not LeTx challenge, in Sprague-Dawley rats is associated with an impaired myocardial function [23]. Hicks et al. found that EdTx at doses of ≤80% lethality rate (LD80) impairs heart function in isolated perfused rat hearts. In contrast, LeTx at a 10-fold greater LD80 dose could achieve comparable effects to EdTx [24]. Additionally, Watson et al. showed that EdTx, but not LeTx challenge, resulted in significantly increased heart rates in rats, and LeTx decreased left ventricular systolic function while EdTx decreased preload [25]. These data indicate LeTx and EdTx may cause cardiac dysfunction through different mechanisms. However, our results showed that LeTx significantly inhibited the cell viability of primary cardiomyocytes, suggesting that LeTx may impair cardiac functions. Our results also revealed that EdTx and LeTx challenge significantly elevated the serum ALT and AST levels in rats, suggesting that EdTx and LeTx impair liver function. In addition, we observed significant renal function indicators in response to EdTx challenge, including BUN, CREA, and phosphorous, consistent with previous reports that EdTx causes kidney lesions [26] and kidney function deterioration [21,27].

Moreover, Comer et al. identified numerous differentially expressed genes in EdTx- and LeTx-treated RAW 264.7 mouse macrophage-like cells, which are involved in inflammation, cell adhesion, immune cell activation, and transcription regulation [28,29]. This study performed microarray and protein–protein network analyses using RNA samples isolated from anthrax=toxin-treated mouse livers to identify potential therapeutic targets against anthrax toxins. A panel of genes associated with anthrax-toxin-induced organ damage was identified and confirmed by qRT-PCR (Figure 3 and Figure 5). LeTx cleaves endogenous MEKs [30], consistent with our findings (Figure S2). On the other hand, Chauncey et al. identified that the MAPKK signaling pathway is the most drastically affected by LeTx [30]. Our study findings suggest that the MAPK pathway plays a critical role in LeTx toxicity.

**Figure 6:**
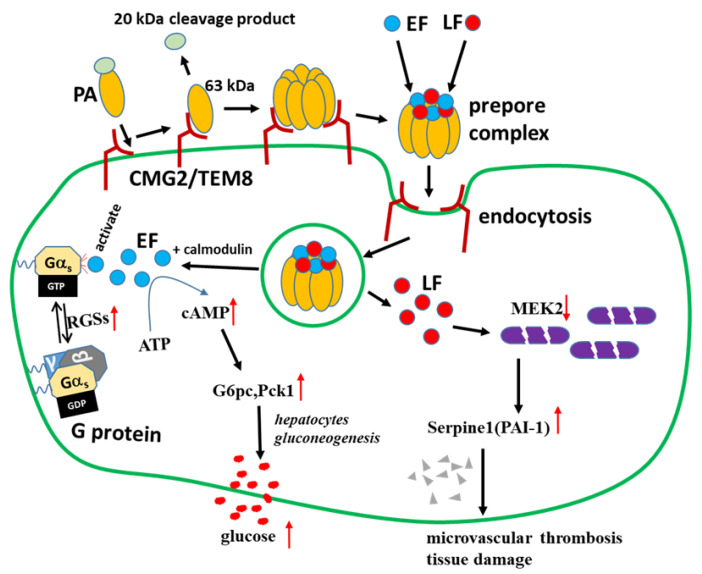
Graphical summary. EdTx consists of EF, a calmodulin-dependent adenylate cyclase that is activated by a GTP-bound Gα subunit. The regulators of G protein signaling (RGSs) are crucial regulatory molecules that influence the nucleotide-bound state of Gα subunits and act as GTPase-activating proteins. The Gα–RGS complex increases the rates of intrinsic GTP hydrolysis to GDP, then EdTx can be activated to convert intracellular ATP to cAMP, resulting in a dramatic increase in cAMP level. On the other hand, G6pc and Pck1 participate in the regulation of hepatic gluconeogenesis and the liver breaks down glycogen to produce blood glucose in a short period of time when exposed to EdTx. A high level of PAI-1 (a serpin protein), a risk factor for thrombosis and atherosclerosis in the liver and serum, is highly implicated in LeTx-induced cytotoxicity by our study. EF, edema factor; LF, lethal factor; PA, protective antigen; CMG2/TEM8, membrane receptor proteins for PA binding.

Anthrax is known to elevate the concentration of blood glucose [31]. However, the mechanism of this is undefined. The current work showed that in EdTx-treated, but not LeTx-treated A/J mice, the blood glucose level rose immediately after EdTx treatment, peaked at 3 h, and declined after that, but remained higher than that of the control group until 9 h after EdTx treatment (Figure 1). These findings suggest that the liver breaks down glycogen quickly when exposed to EdTx, as the liver is the major site of endogenous glucose production [32] and glycogen storage [33]. Intriguingly, the expressions of G6pc, Pck1, and Sik1 were upregulated in primary hepatocytes and livers. G6pc belongs to the glucose-6-phosphatase system. Pck1 plays an important role in gluconeogenesis and stimulates hepatic glucose production [34,35]. Both genes are involved in the LKB1–SIK pathway regulating hepatic gluconeogenesis [36]. Therefore, EdTx may enhance gluconeogenesis and glycogenolysis via the LKB1–SIK pathway in the early stages of anthrax disease, leading to a prompt rise in blood glucose levels (Figure 6).

It has been reported that EdTx causes an increase in the demand for intracellular calcium, leading to a decrease in calcium within the peripheral circulation [37]. The expression of Ramp3, which responds to altered extracellular calcium levels and contributes to calcium homeostasis, is upregulated in this process [38]. This is consistent with our finding that Ramp3 was upregulated in EdTx-treated mouse liver (Figure 3). On the other hand, EdTx consists of edema factor (EF)/adenylate cyclase, an 89-kDa protein secreted by *B. anthracis*, the Gram-positive bacterium responsible for causing anthrax. It is part of a family of bacterial toxins that increase intracellular cyclic AMP (cAMP) levels [7], which can potently influence the conversion of ATP to cAMP in the presence of calmodulin. Therefore, when EF is translocated into the cytosol, EF/calmodulin can increase cAMP in a dose-dependent manner. The regulators of G protein signaling (RGSs) are important regulatory proteins affecting the nucleotide-bound state of Gα subunits, with GTPase-activating functions [39]. The Gα–RGS complex increases the rate of intrinsic GTP hydrolysis to form GDP by about 2000-fold [40]. In this work, *Rgs1* and *Rgs2* levels were both significantly increased by EdTx in primary hepatocytes and liver tissues (Figure 3), resulting in a dramatic increase in the cAMP level, as observed in the animal model (Figure 1 and Figure 6). In addition, an increased level of cAMP is associated with clinical situations that predispose to infections, and the disruption of cAMP generation is a promising therapeutic strategy against these diseases [41,42]. The *Ramp3*, *Pck1*, *G6pc*, *Rgs1*, *Fos*, *Fosl2*, *Hcar2*, *Cxcl2*, and *Cxcl3* genes analyzed in our study are associated with cAMP production. Our findings demonstrated that the knockdown of these genes, individually or in combination, protected primary hepatocytes against the EdTx-mediated elevation of the cAMP level. This suggests that these genes are potential therapeutic targets against anthrax and other infectious diseases.

In the present study, we found that *Rgs1*, *G6pc*, *Fosl2*, *Hcar2*, *Cxcl2*, and *Cxcl3* are also promising targets against the cytotoxicity of EdTx. Although the knockdown of these genes showed a reduced cAMP level, extensive studies in vivo are required to evaluate these genes as gene therapy targets and develop anti-EdTx-based drugs or vaccines. We have also previously reported that the knockdown of the anthrax toxin receptor Cmg2 protects against the increase in intracellular cAMP induced by EdTx [15]. Moreover, in the present study, we found that the mechanism by which LeTx damages liver function differs from that of EdTx. High levels of PAI-1 in the liver and serum were highly implicated in LeTx-associated damage. PAI-1 represents a serine protease inhibitor (serpin) with major inhibitory effects on tissue plasminogen activator (tPA) and urokinase (uPA). PAI-1 is a risk factor for thrombosis and atherosclerosis [43], and decreased PAI-1 amounts resulted in a reduced suppression of fibrinolysis and, conversely, enhanced fibrin degradation [44]. Previous studies have reported that PAI-1 deficiency is characterized by mild-to-moderate bleeding [45] and that PAI-1 deficiency starkly reduces lung fibrin accumulation and highly suppresses inflammatory events and injury, with no impact on upstream coagulation [46]. Our results showed that PAI-1^−/−^ mice are more tolerant to LeTx than WT mice in the early stages of LeTx challenge. High hepatic PAI-1 amounts indicate that PAI-1 plays a crucial role in liver injury. Thus, PAI-1-encoding Serpine1 is a potential therapeutic target against LeTx in the early stage of infection.

There are still some limitations of our study. We elucidated that PAI-1 was one essential target in liver. However, the regulatory mechanisms of PAI-1 should be further revealed. Furthermore, PAI-1 inhibitors, including tiplaxtinin (PAI-039) and three generations of TM-related drugs (TM5001, TM5009, TM5275, TM5441, TM5509, and TM5614), have been used in clinical trials. These inhibitors have been used to study cancer, cardiovascular disease, pulmonary fibrosis renal diseases, liver diseases, and others [47]. The application of these inhibitors may induce some side effects.

## 4. Conclusions

In summary, our findings are valuable for understanding anthrax pathogenesis, providing potential therapeutic targets that may prevent the cytotoxicity induced by anthrax toxins in the liver and the heart. Among these targets, PAI-1 inhibitors may be focused on to further evaluate their safety and potential therapeutical application. A new generation antibiotic-independent, host-targeted therapeutics against anthrax will likely be developed in the near future [48,49].

## 5. Materials and Methods

### 5.1. Preparation of EdTx and LeTx

The EdTx (EF plus PA) and LeTx (LF plus PA) solutions in this study were prepared by dissolving recombinant EF (NR-13413, BEI Resources, Manassas, VA, USA) and LF (NR-4368, BEI Resources) in a culture medium and incubating for 10 min, followed by mixing with a double quantity of recombinant PA (NR-3780, BEI Resources) and incubating for an additional 10 min.

### 5.2. Animal Studies

All animal studies were carried out using the same number of male and female mice whenever possible, following protocols approved by the Animal Care and Use Committee of the Texas Tech University Health Sciences Center, El Paso. In the EdTx challenge experiments, 6 to 8-week-old A/J mice were randomly assigned to 3 groups. Each mouse was administered with 20 μg or 40 μg EdTx (a combination of EF plus PA at 1:2) in 0.1 mL of PBS or with PBS only via intravenous injection. The animal experiment flow chart is shown in Figure 1A. In the LeTx challenge experiments, 6–8-week-old littermate mice (specifically the C57BL/6J strain) were randomly divided into 6 groups. Each mouse was intravenously injected (tail vein injection) with 8.75, 12.5, 18.75, 25, or 50 μg LeTx (a combination of LF plus PA at 1:2) in 0.2 mL of PBS or with PBS only. Five-to-six-week-old PAI-1 knockout (PAI-1^−/−^) C57BL/6J mice were provided by the Jackson Laboratory and intravenously injected with PBS or LeTx, as indicated. All mice were monitored after injection for signs of malaise or mortality until the end of the experiments.

### 5.3. Cell Culture

Primary cardiomyocytes were purified from 1–3-day-old neonatal C57BL/6J mice with the Pierce Primary Cardiomyocyte Isolation Kit (Thermo Fisher Scientific, Waltham, MA, USA), as directed by the manufacturer. Primary hepatocytes were obtained from the livers of 1–3-day-old neonatal A/J mice based on a previous report [50]. Briefly, cell culture plates underwent coating with 0.01% gelatin. Liver specimens were minced (1 mm^3^ pieces), followed by washing with Hank’s balanced salt solution (HBSS, Thermo Fisher Scientific) containing 3 mM CaCl_2_ (Sigma-Aldrich, St. Louis, MO, USA). An equal volume of collagenase H (Roche Life Science, Indianapolis, IN, USA) was supplemented in the solution to achieve 0.08 U. The specimens were incubated at 37 °C for 1 h, followed by washing with chilled hepatocyte wash medium (William’s E Medium; Thermo Fisher Scientific), supplemented with 12% heat-inactivated FBS (Thermo Fisher Scientific), 0.02 VL insulin-transferrin-sodium selenite medium supplement, 30 mM sodium pyruvate (Sigma), penicillin (100 U/mL), and streptomycin (100 μg/mL). After lysis, cell counting was performed, and cells were seeded at 1.5 × 10^6^/well (6-well plates), 8 × 10^5^/well (12-well plates, or 5 × 10^5^/well (24-well plates). These cells were maintained in a hepatocyte culture medium (hepatocyte wash medium supplemented with 5 nM dexamethasone from Sigma-Aldrich and growth factors from human hepatocarcinoma culture medium) in a humid environment with 5% CO_2_ at 37 °C, with the medium refreshed being every other day.

### 5.4. Survival Analysis

Ten mice from each group were randomly selected for Kaplan–Meier survival analysis, and survival curves were plotted.

### 5.5. Blood Glucose Test

Blood was drawn from the tail veins of 10 randomly selected mice from each group at 0, 3, 6, 9, 12, 24, 30, and 36 h after PBS or EdTx injection. This was used for a blood glucose test using a glucose meter (Henry Schein Inc., Melville, NY, USA).

### 5.6. ELISA

Three random mice per group were euthanized at 0, 6, 12, 24, and 36 h after EdTx treatment. Their blood and livers were sampled. Serum was obtained after a 10-min centrifugation of the blood specimens at 3000× *g*. After collection, the liver samples (100 mg) were immediately frozen in liquid nitrogen and then homogenized using a stainless-steel mortar and pestle into fine powder. The cAMP levels in the serum and liver were assessed with a cAMP ELISA kit (Enzo Life Sciences, Farmingdale, NY, USA), as directed by the manufacturer.

For the in vitro study, primary hepatocytes underwent seeding in 12-well plates at 3 × 10^5^/well and were grown overnight. Cells were then administered with 0, 0.25, 0.5, 1, 2, or 4 μg/mL of EdTx, followed by lysis with 0.5 mL of 0.1 M HCl at 0, 0.25, 0.5, 1, 2, 4, 6, 8, 16, or 24 h post-treatment for 10 min. The cAMP concentrations in the cell lysates were measured with the above cAMP ELISA kit [51].

For PAI-I detection, C57BL/6J (wild type, PAI-1^+/+^, n = 5) and PAI-1^−/−^ (C57BL/6J background, n = 5) mice were challenged intravenously with 50 μg LeTx in 0.2 mL of PBS. Blood sample collection occurred 24 h post-injection. According to the manufacturer’s protocol, the PAI-1 levels in the mouse serum were measured with the Mouse PAI-1 ELISA Kit (Thermo Fisher Scientific).

### 5.7. Flow Cytometry Detection of Anthrax Toxin Receptors

Primary hepatocytes and cardiomyocytes were collected and washed with PBS supplemented with 2% FBS. Cell staining was performed with primary rabbit polyclonal anti-TEM8 antibodies (Abcam, Cambridge, UK) and secondary donkey anti-rabbit IgG PE (Affymetrix eBioscience, San Diego, CA, USA), and/or primary goat polyclonal anti-CMG2 (Abcam) and secondary chicken anti-goat Alexa Fluor 488 antibodies (Thermo Fisher Scientific). Data analysis utilized a BD FACS Canto™ II flow cytometer (BD Biosciences, San Jose, CA, USA) with FlowJo v7.6.5 or vX.0.6 (Tree Star, Ashland, OR, USA).

### 5.8. siRNA Transfections

siRNAs targeting the murine Ramp3 (si-Ramp3), Rgs1 (si-Rgs1), Pck1 (si-Pck1), G6pc (si-G6pc), Hcar2 (si-Hcar2), Fosl2 (si-Fosl2), Fos (si-Fos), Cxcl2 (si-Cxcl2), Cxcl3 (si-Cxcl3), and Cmg2 (si-CMG2) genes were provided by Santa Cruz Biotechnology. The sequence information is shown in Supplementary Table S4. si-GFP was used as a nonspecific control. Primary hepatocytes underwent seeding in 24-well plates at 1.5 × 10^5^/well in 0.3 mL of Opti-MEM^®^ medium (Thermo Fisher Scientific) and were grown for 5–7 days prior to transient transfection with 50 pmol siRNA using Lipofectamine RNAiMax (Thermo Fisher Scientific), as suggested by the manufacturer. The cells were then cultured for 48 h and transfected again, followed by an additional incubation of 48 h.

### 5.9. MTT Assay

Cells underwent seeding in 24-well or 96-well plates, and were administered with 4 µg/mL of EdTx or 2 μg/mL of LeTx for the indicated times, with PBS used as a negative control. MTT solution (5 mg/mL) was supplemented for 2 h at 37 °C. After medium removal, dimethyl sulfoxide (0.2 mL/well in 96-well plates and 0.5 mL/well in 24-well plates) was used to dissolve formazan crystals. A PowerWave XS2 spectrophotometer (BioTek, Shoreline, WA, USA) was utilized for absorbance reading at 570 nm, and the data were normalized to the viable cells in the control group.

### 5.10. Mitochondria-Regulated Apoptosis Assay by JC-1 Staining

Mitochondrial membrane potential was assessed with the JC-1 (5,5′,6,6′-tetrachloro-1,1′,3,3′-tetraethylbenzimidazolycarbocyanine iodide) kit (Thermo Fisher Scientific), as described by the manufacturer. A Nikon ECLIPSE Ti inverted fluorescence microscope equipped with a digital CMOS camera (magnification, ×20) was utilized for imaging, and image analysis employed the NIS-Element software (Nikon, Tokyo, Japan).

### 5.11. Western Blot Assay

The hearts and livers were collected from three randomly selected mice in each group at 24 h after the administration of 50 μg of LeTx. All cells were seeded in 6-well plates and administered with 2 mL of 2 µg/mL LeTx for 0, 2, 6, 12, or 18 h. Cells and organs were lysed with cell lysis buffer and RIPA buffer (Cell Signaling Technology, Danvers, MA, USA), respectively, containing 1 μM PMSF. Total protein (30 µg) was resolved by 10% SDS-PAGE, followed by transfer onto nitrocellulose membranes with a semi-dry transblot apparatus (Bio-Rad, Hercules, CA, USA). Membranes were next blocked using 5% skimmed milk in PBS with 1% Tween (PBST) for 1 h, followed by successive incubations with mouse monoclonal anti-MEK2 (Santa Cruz Biotechnology, Dallas, TX, USA) or anti-β-actin (Cell Signaling Technology) antibody (4 °C overnight) and alkaline phosphatase-linked anti-mouse IgG (ambient, 1 h). Following PBST rinses, chemiluminescent signals were obtained using 5-bromo-4-chloro-3-indolyl phosphate/nitro blue tetrazolium substrate (Sigma).

### 5.12. Indocyanine Green (ICG) Uptake-and-Efflux Assay

Primary hepatocytes or cardiomyocytes were administered with 2 μg/mL of EdTx or LeTx for 6 h followed by a 2 h incubation with DMSO-dissolved ICG (final concentration, 0.5 mg/mL; Sigma). The cells were further monitored for 24 h in a culture medium prior to the measurement of ICG efflux [52]. A Nikon ECLIPSE Ti inverted microscope was employed for imaging.

### 5.13. Blood Chemistry Analysis

Four mice were randomly selected from each group, and the serum samples were collected at 18 h after injection and sent to IDEXX BioResearch (West Sacramento, CA, USA) for blood chemistry analysis.

### 5.14. Histology Analyses

Three random mice were selected per group, and the liver of each mouse was harvested at 18 h after injection. The liver tissue sample was fixed with 4% paraformaldehyde, followed by the preparation of paraffin-embedded tissue sections at 4 μm, which were then stained by the H&E and periodic acid–Schiff (PAS) methods before being sent to Duke University Medical Center for a blinded histology analysis by independent pathologists.

### 5.15. Intracellular PAS Staining

PAS staining was applied to detect glycogen in primary hepatocytes and cardiomyocytes. The cells underwent seeding on 8-well chamber slides (Thermo Fisher Scientific) and treatment with 4 μg/mL of EdTx or 2 μg/mL of LeTx for 6 h, followed by fixation with formaldehyde and staining with PAS using a PAS staining system (Sigma) or a Glycogen Colorimetric Assay Kit II (BioVision, Milpitas, CA, USA), using the respective manufacturers’ protocols.

### 5.16. Microarray

Primary hepatocytes and cardiomyocytes were exposed to 4 µg/mL of EdTx for 6 h and 2 μg/mL of LeTx for 18 h, with PBS as a negative control. The cells were incubated in William’s E medium and examined in quadruplicate. Total RNA extraction from the cells was performed, and the samples were sent to the Genomics & Microarray Core Facility at University of Texas Southwestern Medical Center in Dallas for microarrays employing the GeneChips Mouse Transcriptome Assay 1.0 (Affymetrix). Partek Genomic Suite (Partek, St. Louis, MO, USA) was utilized for data analysis.

### 5.17. Quantitative Real-Time PCR (qRT-PCR)

At 90 to 95% confluence in 6-well plates, cells were administered with 4 µg/mL of EdTx for 6 h or 2 μg/mL of LeTx for 18 h. The livers and hearts were collected from mice challenged with 20 µg EdTx for 18 h or 50 μg LeTx for 24 h. Total RNA isolation from cell or tissue samples used the RNeasy Mini kit (Qiagen, San Diego, CA, USA), and the ProtoScript^®^ First Strand cDNA Synthesis Kit (New England BioLabs, Ipswich, MA, USA) was employed for reverse transcription, as per the manufacturer’s instructions. Murine Tem8 (584 bp), Cmg2 (364 bp), and Gapdh (239 bp) fragments were amplified with Master Mix, and all primers are listed in Supplementary Table S6, as previously described [11].

### 5.18. Statistical Analysis

For the microarray experiment, data are presented as mean ± standard deviation, and Partek v6.6 was utilized for data analysis. A two-tailed paired *t*-test was used to compare the expression levels. For other assays, GraphPad Prism v5.04 was used for data analysis. A two-tailed, one-way analysis of variance with post hoc Dunnett’s multiple comparison test was employed for multiple group comparisons. *p* < 0.05 indicates statistical significance.

## Data Availability

The original microarray data presented in the study are openly available in the Gene Expression Omnibus (GEO) (accession number GSE116755) at https://www.ncbi.nlm.nih.gov/geo/query/acc.cgi?acc=gse116755 (accessed on 16 January 2025).

## Supplementary Materials

The following supporting information can be downloaded at: https://www.mdpi.com/article/10.3390/toxins17020054/s1, Figure S1: Protein–protein network analysis among the 218 genes which were found to have significant expression changes in primary hepatocytes exposed to EdTx treatment compared with PBS-treated cells; Figure S2: The in vivo toxicity of anthrax LeTx. (A) Survival curve of wild type (WT) C57BL/6J mice challenged with 0, 8.75, 12.5, 18.75, 25, or 50 µg of LeTx in 0.2 mL of PBS. (B) Western blot assay for cleaved MEK2 and β-actin expression in the heart and liver of WT C57BL/6J mice treated with 50 μg of LeTx for 24 h; Figure S3: The mRNA expression of anthrax toxin receptors in primary hepatocytes and cardiomyocytes, liver, and heart tissues of mice; Figure S4: Cell viability of primary cardiomyocytes treated with a single dose of LeTx; Table S1: The effect of anthrax toxins on blood chemistry in mice; Table S2: EdTx-mediated effects on the expression of genes in mouse primary hepatocytes and livers; Table S3: Efficiency of gene knockdown in mouse primary hepatocytes after EdTx challenge; Table S4: Sequence information of siRNA used in this study; Table S5: Primers used for qPCR in this paper; Table S6: LeTx-mediated effects on the expression of selected genes in mouse primary cardiomyocytes, HL-1 cells, primary hepatocytes, mouse livers, and mouse heart.

## Abbreviations

ALB: albumin; ALP: alkaline phosphatase; ALT: alanine aminotransferase; AST: aspartate aminotransferase; BUN: blood urea nitrogen; CK: creatine kinase; CMG2: capillary morphogenesis protein 2; CREA: creatinine; EdTx: edema toxin; EF: edema factor; GEO: Gene Expression Omnibus; GLB: globulin; ICG: indocyanine green; LD80: 80% lethality rate; LeTx: lethal toxin; LF: lethal factor; PA: protective antigen; PAS: periodic acid–Schiff; PBST: PBS containing 1% Tween; siRNA: small interfering RNA; TEM8: tumor endothelium marker 8; TP: total protein; tPA: tissue plasminogen activator; uPA: urokinase plasminogen activator.
