# Supplementary material for: Identification of Potential Therapeutic Targets Against Anthrax-Toxin-Induced Liver and Heart Damage
Source: Toxins (Basel). 2025 Jan 24;17(2):54. doi: 10.3390/toxins17020054 (PMC11861023; doi:10.3390/toxins17020054)
Supplement: Supplementary file 1 [file toxins-17-00054-s001.zip › Supplementary Figures S1-S4.pdf]

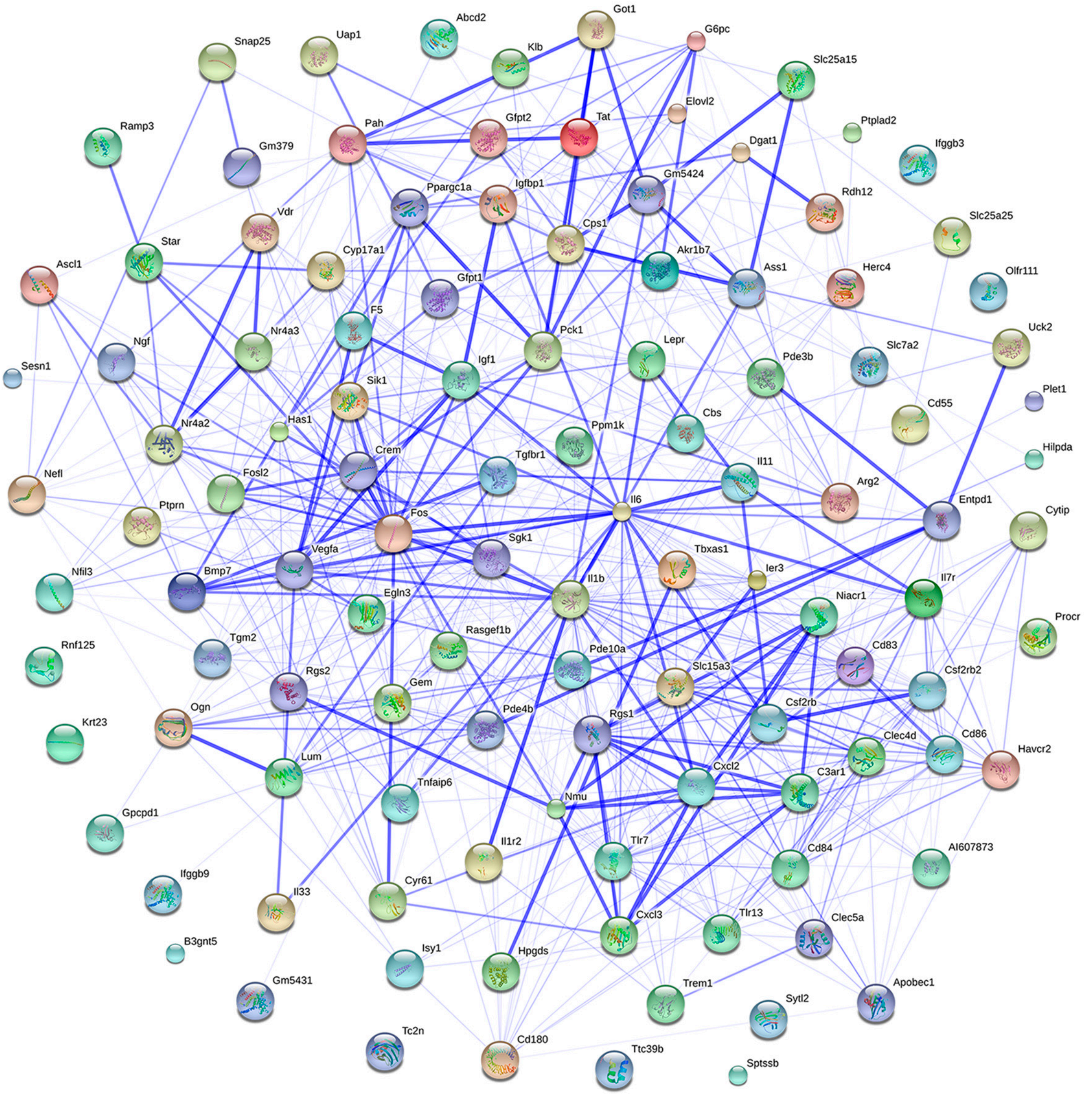

Figure S1: Protein–protein network analysis among the 218 genes which were found to have significant expression changes in primary hepatocytes exposed to EdTx treatment compared with PBS-treated cells.

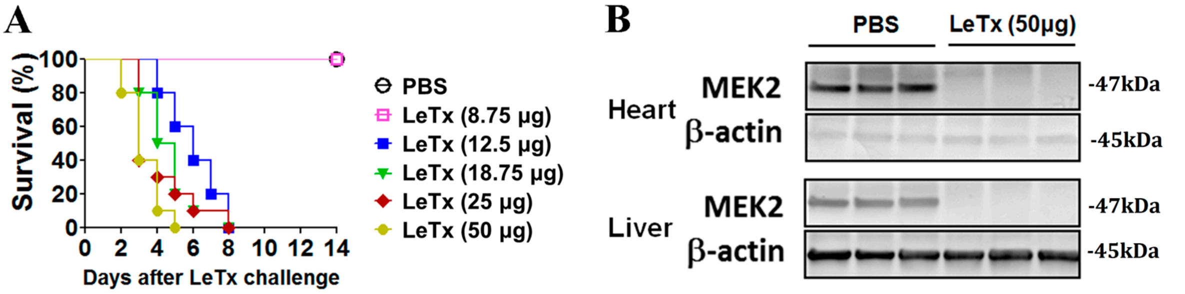

Figure S2: The in vivo toxicity of anthrax LeTx. (A) Survival curve of wild type (WT) C57BL/6J mice challenged with 0, 8.75, 12.5, 18.75, 25, or 50 μg of LeTx in 0.2 mL of PBS. (B) Western blot assay for cleaved MEK2 and β-actin expression in the heart and liver of WT C57BL/6J mice treated with 50 μg of LeTx for 24 h.

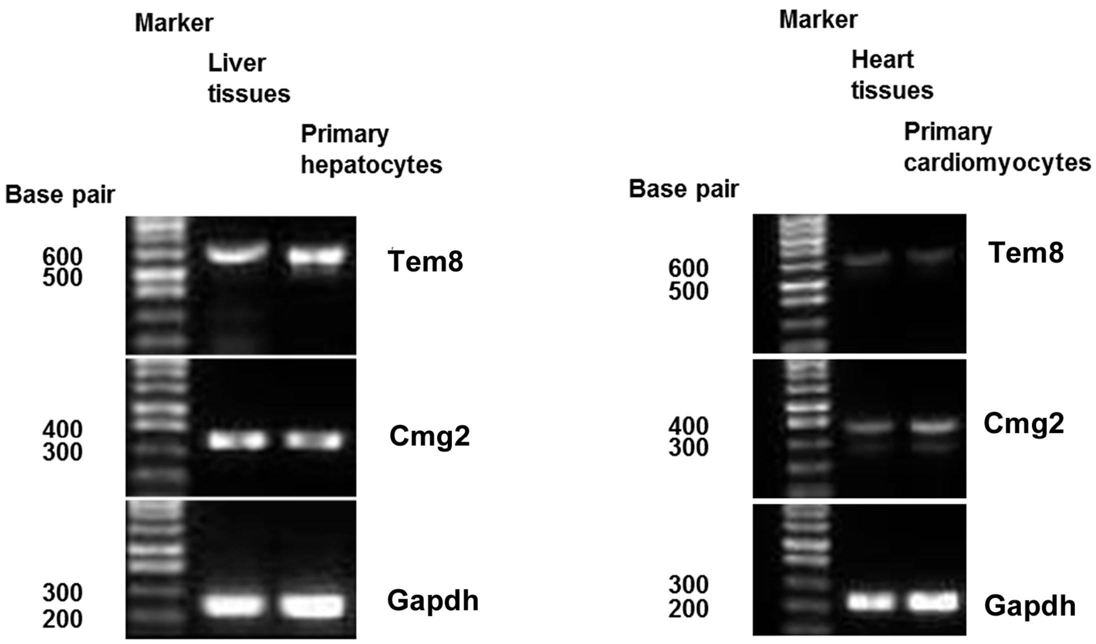

Figure S3 : The mRNA expression of anthrax toxin receptors in primary hepatocytes and cardiomyocytes, liver, and heart tissues of mice.

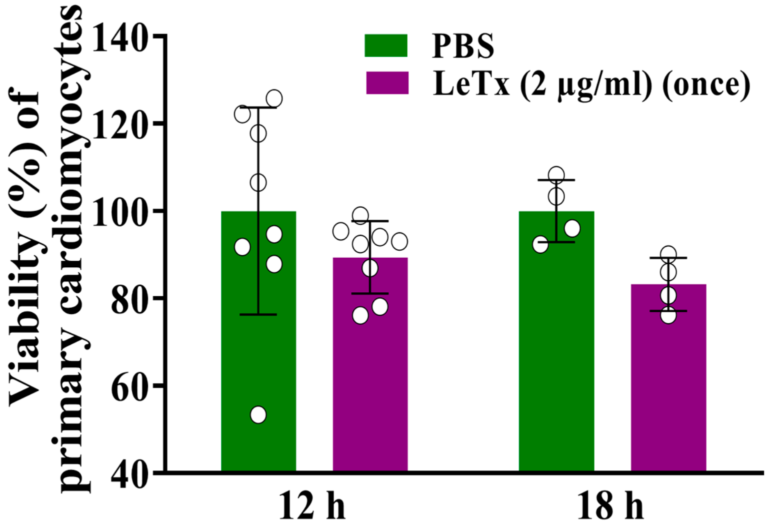

Figure S4: Cell viability of primary cardiomyocytes treated with a single dose of LeTx;.
