# Supplementary material for: Identification of Potential Therapeutic Targets Against Anthrax-Toxin-Induced Liver and Heart Damage
Source: Toxins (Basel). 2025 Jan 24;17(2):54. doi: 10.3390/toxins17020054 (PMC11861023; doi:10.3390/toxins17020054)
Supplement: Supplementary file 1 [file toxins-17-00054-s001.zip › Supplementary Table S1.pdf]

Supplementary Table S1. The effect of anthrax toxins on blood chemistry in mice

**Blood Chemistry**	**Unit**	**PBS (A/J)**	**20µg EdTx (A/J)**	**PBS (Balb/c)**	**50µg LeTx (Balb/c)**
Alanine Aminotransferase (ALT/SGPT)	U/L	56.25 ± 22.90	242.60 ± 37.79**	42.75 ± 8.88	172.50 ± 40.12**
Aspartate Aminotransferase (AST/SGOT)	U/L	141.75 ± 42.30	281.00 ± 24.19**	66.75 ± 8.62	398.00 ± 162.01**
Albumin (ALB)	g/dL	3.08 ± 0.10	2.74 ± 0.21*	2.68 ± 0.05	1.13 ± 0.30**
Globulin	g/dL	1.90 ± 0.08	1.58 ± 0.08**	2.25 ± 0.06	1.00 ± 0.28**
Albumin / Globulin (A/G)		1.63 ± 0.10	1.74 ± 0.15	1.20 ± 0.00	1.15 ± 0.13
Alkaline Phosphatase (ALP)	U/L	157.75 ± 40.42	240.40 ± 31.20*	163.50 ± 18.64	142.00 ± 4.54
Bicarbonate (TCO_2)	mmol/L	19.00 ± 3.56	10.80 ± 4.02*	22.00 ± 1.63	17.25 ± 7.75*
Blood Urea Nitrogen (BUN)	mg/dL	25 ± 4.69	118.80 ± 20.86**	30.25 ± 1.26	90.25 ± 36.70*
BUN: Creatinine ratio(B/C)		125.00 ± 23.45	180.66 ± 46.95	302.50 ± 12.58	546.67 ± 270.06
Calcium (Ca)	mg/dL	9.40 ± 0.18	6.88 ± 0.58**	9.78 ± 0.17	7.75 ± 0.51**
Chloride (Cl)	mmol/L	117.50 ± 1.73	113.00 ± 3.16*	112.50 ± 3.00	120.50 ± 3.11*
Cholesterol (CHOL)	mg/dL	78 ± 5.48	41.20 ± 3.19**	125.50 ± 9.00	34.50 ± 11.00**
Creatine Kinase (CK)	U/L	1699.25 ± 1108.74	7300.00 ± 3210.72*	616.50 ± 109.42	1960.50 ± 980.46*

Creatinine (CREA)	mg/dL	0.20 ± 0.00	0.68 ± 0.16**	0.10 ± 0.00	0.10 ± 0.08
Phosphorous (P)	mg/dL	8.80 ± 1.03	19.94 ± 0.91**	9.08 ± 1.02	9.30 ± 1.68
Calcium: Phosphorous (Ca/P)		1.08 ± 0.13	0.35 ± 0.04**	1.09 ± 0.14	0.85 ± 0.15
Potassium (K)	mmol/L	6.20 ± 0.54	9.40 ± 0.87**	6.25 ± 0.42	6.38 ± 1.25
Sodium (Na)	mmol/L	154.25 ± 2.36	150 ± 3.40	154.50 ± 1.91	153.75 ± 0.96
Sodium: Potassium Ratio (Na/K)		25.00 ± 2.45	16.00 ± 1.973**	24.81 ± 1.74	24.73 ± 4.15
Total Protein (TP)	g/dL	4.98 ± 0.10	4.32 ± 0.21**	4.93 ± 0.10	2.13 ± 0.57**

Blood samples from all mice were collected at 18 hours after EdTx (20 µg/per mouse) or 24 hours after LeTx (50 µg/per mouse) challenging.

The mean ± SD. for 4 replicates (n=4) from each group. $^{*}p < 0.05$ versus PBS group; $^{**}p < 0.01$ versus PBS group.
