# Supplementary material for: Identification of Potential Therapeutic Targets Against Anthrax-Toxin-Induced Liver and Heart Damage
Source: Toxins (Basel). 2025 Jan 24;17(2):54. doi: 10.3390/toxins17020054 (PMC11861023; doi:10.3390/toxins17020054)
Supplement: Supplementary file 1 [file toxins-17-00054-s001.zip › Supplementary Table S2.pdf]

**Supplementary Table S2. EdTx-mediated effects on the expression of genes in mouse primary hepatocytes and livers**

| Gene name (218) | Gene bank ID | Mouse primary hepatocytes | | Mouse Livers |
|---|---|---|---|---|
| | | Microarray (log ratio) | qPCR (fold change) | qPCR (fold change) |
| Amino Acid Metabolism (9) | | | | |
| Mus musculus arginase type II (Arg2) | NM_009705 | 4.311* | 27.939* | 28.806* |
| Mus musculus argininosuccinate synthetase 1 (Ass1) | NM_007494 | 2.310** | 3.17 | 1.062 |
| Mus musculus cystathionine beta-synthase (Cbs), transcript variant 3 | NM_001271353 | 2.557* | 5.854 | 0.454* |
| Mus musculus carbamoyl-phosphate synthetase 1 (Cps1) | NM_001080809 | 2.186* | 11.582** | 0.945 |
| Mus musculus glutamate oxaloacetate transaminase 1, soluble (Got1) | NM_010324 | 2.118* | 2.957* | 6.584** |
| Mus musculus phenylalanine hydroxylase (Pah) | NM_008777 | 3.618 | 4.021* | 0.609 |
| Mus musculus tyrosine aminotransferase (Tat) | NM_146214 | 7.949* | 34.729** | 8.537* |
| Mus musculus glutamine fructose-6-phosphate transaminase 1 (Gfpt1) | NM_013528 | 2.720** | 1.622 | 0.858 |
| Mus musculus glutamine fructose-6-phosphate transaminase 2 (Gfpt2) | NM_013529 | 3.056** | 4.686** | 8.546** |
| Glucose Metabolism (30) | | | | |

| | | | | |
|---|---|---|---|---|
| Mus musculus aldo-keto reductase family 1, member B7 (Akr1b7) | NM_009731 | 3.731** | 9.450** | 12.204* |
| Mus musculus UDP-GlcNAc:betaGal beta-1,3-N-acetylglucosaminyltransferase 5 (B3gnt5), transcript variant 1 | NM_001159407 | 2.472** | 0.684 | 2.061 |
| Mus musculus cAMP responsive element modulator (Crem), transcript variant 4 | NM_001110850 | 2.664** | 1.431 | 1.04 |
| Mus musculus colony stimulating factor 2 receptor, beta, low-affinity (granulocyte-macrophage) (Csf2rb) | NM_007780 | 2.049* | 3.004** | 2.169* |
| Mus musculus colony stimulating factor 2 receptor, beta 2, low-affinity (granulocyte-macrophage) (Csf2rb2), transcript variant 2 | NM_001287389 | 2.243* | 2.159 | 1.041 |
| Mus musculus cytochrome P450, family 17, subfamily a, polypeptide 1 (Cyp17a1), mRNA | NM_007809 | 5.090* | 4.367** | 8.945 |
| Mus musculus diacylglycerol O-acyltransferase 1 (Dgat1) | NM_010046 | 2.821** | 3.342** | 1.027 |
| Mus musculus ectonucleoside triphosphate diphosphohydrolase 1 (Entpd1) | NM_009848 | 2.625* | 5.781 | 0.389* |
| Mus musculus ethanolamine phosphate phospholyase (Etnppl), transcript variant 2 | NM_001163587 | 3.003* | 13.436 | 1.816 |
| Mus musculus glucose-6-phosphatase, catalytic (G6pc) | NM_008061 | 9.942** | 15.496* | 3.395* |
| Mus musculus GTP binding protein (gene overexpressed in skeletal muscle) (Gem) | NM_010276 | 2.322* | 3.356 | 2.211 |

| | | | | |
|---|---|---|---|---|
| Mus musculus hematopoietic prostaglandin D synthase (Hpgds) | NM_019455 | 0.465** | 0.39 | 1.052 |
| Mus musculus leptin receptor (Lepr), transcript variant 3 | NM_001122899 | 2.258* | 2.051 | 2.126 |
| Mus musculus phosphoenolpyruvate carboxykinase 1, cytosolic (Pck1) | NM_011044 | 4.020* | 42.022** | 3.935* |
| Mus musculus phosphodiesterase 3B, cGMP-inhibited (Pde3b) | NM_011055 | 2.010* | 3.044** | 0.523 |
| Mus musculus phosphodiesterase 4B, cAMP specific (Pde4b) | NM_001177980 | 2.059** | 5.780** | 3.734* |
| Mus musculus peroxisome proliferative activated receptor, gamma, coactivator 1 alpha (Ppargc1a), transcript variant 1 | NM_008904 | 3.345** | 0.637 | 1.603 |
| Mus musculus protein tyrosine phosphatase, receptor type, N (Ptprn) | NM_008985 | 2.077* | 3.130* | 3.954* |
| Mus musculus receptor (calcitonin) activity modifying protein 3 (Ramp3) | NM_019511 | 3.449** | 20.345* | 9.753* |
| Mus musculus retinol dehydrogenase 12 (Rdh12) | NM_030017 | 2.524** | 4.434** | 9.536** |
| Mus musculus regulator of G-protein signaling 1 (Rgs1) | NM_015811 | 6.364** | 15.230* | 21.550* |
| Mus musculus regulator of G-protein signaling 2 (Rgs2) | NM_009061 | 2.205** | 3.106* | 2.363* |
| Mus musculus serum/glucocorticoid regulated kinase 1 (Sgk1) | NM_001161845 | 2.375** | 6.049* | 3.990* |
| Mus musculus salt inducible kinase 1 (Sik1) | NM_010831 | 3.464** | 3.944* | 8.057** |

| | | | | |
|---|---|---|---|---|
| Mus musculus solute carrier family 25 (mitochondrial carrier, phosphate carrier), member 25 (Slc25a25) | NM_001164357 | 2.593** | 3.403** | 4.928* |
| Mus musculus thromboxane A synthase 1, platelet (Tbxas1) | NM_011539 | 0.496* | 0.085** | 0.303* |
| Mus musculus transforming growth factor, beta receptor I (Tgfbr1) | NM_009370 | 2.065** | 1.808 | 0.608 |
| Mus musculus transglutaminase 2, C polypeptide (Tgm2) | NM_009373 | 2.419** | 3.422* | 7.330* |
| Mus musculus UDP-N-acetylglucosamine pyrophosphorylase 1 (Uap1) | NM_133806 | 3.069** | 2.926 | 0.87 |
| Mus musculus uridine-cytidine kinase 2 (Uck2) | NM_030724 | 2.112** | 3.309* | 4.623 |
| Inflammation and Apoptosis (31) | | | | |
| Mus musculus bone morphogenetic protein 7 (Bmp7) | NM_007557 | 2.182* | 7.021* | 0.839 |
| Mus musculus complement component 3a receptor 1 (C3ar1) | NM_009779 | 0.405* | 0.257 | 0.948 |
| Mus musculus CD180 antigen (Cd180) | NM_008533 | 0.251** | 0.051 | 0.793 |
| Mus musculus CD55 antigen (Cd55) | NM_010016 | 2.243** | 4.209 | 0.387** |
| Mus musculus CD86 antigen (Cd86) | NM_019388 | 2.021** | 2.673 | 0.605 |
| Mus musculus chemokine (C-X-C motif) ligand 2 (Cxcl2) | NM_009140 | 3.897** | 9.148* | 47.234** |

| | | | | |
|---|---|---|---|---|
| Mus musculus chemokine (C-X-C motif) ligand 3 (Cxcl3) | NM_203320 | 2.694$^{*}$ | 30.073$^{*}$ | 57.163$^{*}$ |
| Mus musculus cysteine rich protein 61 (Cyr61) | NM_010516 | 0.388$^{*}$ | 0.149$^{**}$ | 1.609 |
| Mus musculus egl-9 family hypoxia-inducible factor 3 (Egln3) | NM_028133 | 3.045$^{**}$ | 5.595$^{**}$ | 0.822 |
| Mus musculus coagulation factor V (F5) | NM_007976 | 2.061$^{*}$ | 6.505$^{**}$ | 7.324$^{*}$ |
| Mus musculus FBJ osteosarcoma oncogene (Fos) | NM_010234 | 3.521$^{*}$ | 3.402$^{**}$ | 44.446$^{*}$ |
| Mus musculus fos-like antigen 2 (Fosl2) | NM_008037 | 2.162$^{**}$ | 3.398$^{**}$ | 5.561$^{*}$ |
| Mus musculus hydroxycarboxylic acid receptor 2 (Hcar2) | NM_030701 | 2.371$^{**}$ | 2.935$^{*}$ | 35.325$^{*}$ |
| Mus musculus hypoxia inducible lipid droplet associated (Hilpda) | NM_001190461 | 3.106$^{**}$ | 5.851$^{*}$ | 28.508$^{**}$ |
| Mus musculus insulin-like growth factor 1 (Igf1) | NM_001111274 | 0.482$^{*}$ | 0.373$^{**}$ | 0.435$^{**}$ |
| Mus musculus interleukin 11 (Il11) | NM_008350 | 2.535$^{*}$ | 12.214$^{*}$ | 4.905$^{*}$ |
| Mus musculus interleukin 1 beta (Il1b) | NM_008361 | 13.017$^{**}$ | 21.006$^{*}$ | 4.225$^{*}$ |
| Mus musculus interleukin 1 receptor, type II (Il1r2) | NM_010555 | 3.212$^{**}$ | 5.765$^{**}$ | 76.847$^{*}$ |
| Mus musculus interleukin 33 (Il33), transcript variant 1 | NM_001164724 | 5.614$^{**}$ | 3.925$^{*}$ | 2.605 |
| Mus musculus interleukin 6 (Il6) | NM_031168 | 4.242$^{**}$ | 1.548 | 3.711 |

| | | | | |
|---|---|---|---|---|
| Mus musculus interleukin 7 receptor (Il7r) | NM_008372 | 2.036* | 4.15 | 1.768 |
| Mus musculus nerve growth factor (Ngf), transcript variant 2 | NM_001112698 | 2.220* | 4.430* | 1.56 |
| Mus musculus nuclear receptor subfamily 4, group A, member 2 (Nr4a2) | NM_001139509 | 6.330* | 8.035* | 4.372** |
| Mus musculus nuclear receptor subfamily 4, group A, member 3 (Nr4a3) | NM_015743 | 4.432* | 2.943* | 4.231** |
| Mus musculus protein C receptor, endothelial (Procr) | NM_011171 | 2.473* | 8.688 | 1.755 |
| Mus musculus RasGEF domain family, member 1B (Rasgef1b) | NM_145839 | 2.210* | 3.493* | 8.485** |
| Mus musculus steroidogenic acute regulatory protein (Star) | NM_011485 | 3.116* | 9.675* | 1.125 |
| Mus musculus toll-like receptor 7 (Tlr7), transcript variant 3 | NM_133211 | 0.309* | 0.237 | 0.795 |
| Mus musculus tumor necrosis factor alpha induced protein 6 (Tnfaip6) | NM_009398 | 2.886** | 3.496* | 8.212* |
| Mus musculus triggering receptor expressed on myeloid cells 1 (Trem1) | NM_021406 | 6.643* | 7.024* | 6.629** |
| Mus musculus vascular endothelial growth factor A (Vegfa), transcript variant 1 | NM_001025250 | 2.085** | 3.234* | 0.599 |
| Others (55) | | | | |
| Gm20412: havana: putative chromosome | ENSMUST00000173249 | 2.74** | | |
| Mus musculus achaete-scute complex homolog 1 (Drosophila) (Ascl1) | NM_008553 | 5.083** | | |

| | | |
|---|---|---|
| Mus musculus microRNA 223 (Mir223) | NR_029801 | 0.444$^{**}$ |
| Mus musculus RIKEN cDNA 9930111J21 gene 1 (9930111J21Rik1) | NM_001114679 | 0.365$^{**}$ |
| Mus musculus protein phosphatase 1K (PP2C domain containing) (Ppm1k) | NM_175523 | 2.400$^{**}$ |
| Mus musculus RIKEN cDNA 9930111J21 gene 2 (9930111J21Rik2) | NM_173434 | 0.359$^{*}$ |
| Mus musculus klotho beta (Klb) | NM_031180 | 2.023$^{**}$ |
| Mus musculus ATP-binding cassette, sub-family D (ALD), member 2 (Abcd2) | NM_011994 | 0.471$^{**}$ |
| Mus musculus expressed sequence AI607873 (AI607873) | NM_001204910 | 0.372$^{**}$ |
| Mus musculus hyaluronan synthase1 (Has1) | NM_008215 | 2.396$^{**}$ |
| Gm5424: ensembl:known chromosome | ENSMUST00000053865 | 2.547$^{**}$ |
| Mus musculus cytohesin 1 interacting protein (Cytip) | NM_139200 | 5.391$^{**}$ |
| Mus musculus synaptotagmin-like 2 (Sytl2), transcript variant 2 | NM_001040085 | 2.817$^{**}$ |
| Mus musculus apolipoprotein B mRNA editing enzyme, catalytic polypeptide 1 (Apobec1), transcript variant 2 | NM_001134391 | 0.342$^{**}$ |
| Mus musculus predicted gene 5431 (Gm5431) | NM_001024230 | 0.434$^{**}$ |

| | | |
|---|---|---|
| Gm15675: havana: known chromosome | ENSMUST00000130486 | 2.246** |
| Mus musculus sestrin 1 (Sesn1), transcript variant 2 | NM_001013370 | 0.482** |
| Mus musculus neurofilament, light polypeptide (Nefl) | NM_010910 | 2.259** |
| Gm24060: ncrna: known chromosome | ENSMUST00000157494 | 2.245** |
| Mus musculus glycerophosphocholine phosphodiesterase GDE1 homolog (S. cerevisiae) (Gpcpd1), transcript variant 3 | NM_001042671 | 2.071** |
| Gm379: ensembl: known chromosome | ENSMUST00000178160 | 2.263** |
| Mus musculus placenta expressed transcript 1 (Plet1) | NM_029639 | 2.172** |
| Mus musculus vitamin D receptor (Vdr) | NM_009504 | 2.156** |
| Mus musculus solute carrier family 15, member 3 (Slc15a3) | NM_023044 | 2.250** |
| Mus musculus neuromedin U (Nmu) | NM_019515 | 2.005** |
| Mus musculus synaptosomal-associated protein 25 (Snap25), transcript variant 1 | NM_011428 | 2.090** |
| Mus musculus tetratricopeptide repeat domain 39B (Ttc39b) | NM_027238 | 2.077** |
| Gm27031: havana: known chromosome | ENSMUST00000181988 | 2.029** |

| | | |
|---|---|---|
| Mus musculus serine palmitoyltransferase, small subunit B (Sptssb), transcript variant 1 | NM_001164210 | 3.770** |
| Mus musculus CD83 antigen (Cd83), transcript variant 1 | NM_001289915 | 2.253* |
| Mus musculus hect domain and RLD 4 (Herc4), transcript variant 2 | NM_026101 | 2.200* |
| Mus musculus C-type lectin domain family 5, member a (Clec5a), transcript variant 1 | NM_001038604 | 0.425* |
| Mus musculus immediate early response 3 (Ier3) | NM_133662 | 2.024* |
| Mus musculus phosphodiesterase 10A (Pde10a), transcript variant 2 | NM_011866 | 2.241* |
| Mus musculus ring finger protein 125 (Rnf125) | NM_026301 | 2.789* |
| Mus musculus ISY1 splicing factor homolog (S. cerevisiae) (Isy1) | NM_133934 | 2.157* |
| Mus musculus solute carrier family 7 (cationic amino acid transporter, y+ system), member 2 (Slc7a2), transcript variant 2 | NM_001044740 | 3.455* |
| Mus musculus solute carrier family 25 (mitochondrial carrier ornithine transporter), member 15 (Slc25a15) | NM_181325 | 2.465* |
| PREDICTED: Mus musculus uncharacterized LOC100503338 (LOC100503338), ncRNA | XR_106025 | 0.412* |
| Mus musculus hepatitis A virus cellular receptor 2 (Havcr2) | NM_134250 | 2.201* |

| | | |
|---|---|---|
| Mus musculus microRNA 493 (Mir493) | NR_030573 | 2.108* |
| Mus musculus olfactory receptor 111 (Olfr111) | NM_001005485 | 0.388* |
| Mus musculus keratin 23 (Krt23) | NM_033373 | 2.267* |
| Mus musculus toll-like receptor 13 (Tlr13) | NM_205820 | 0.358* |
| n-R5s54: ncrna: known chromosome | ENSMUST00000083837 | 3.603* |
| Mus musculus uncharacterized LOC102631757 (LOC102631757), transcript variant 2, long non-coding RNA | NR_110502 | 2.096* |
| Mus musculus nuclear factor, interleukin 3, regulated (Nfil3) | NM_017373 | 2.113* |
| Mus musculus protein tyrosine phosphatase-like A domain containing 2 (Ptplad2) | NM_025760 | 0.420* |
| Mus musculus insulin-like growth factor binding protein 1 (Igfbp1) | NM_008341 | 2.372* |
| Mus musculus tandem C2 domains, nuclear (Tc2n), transcript variant 2 | NM_001082976 | 2.177* |
| Mus musculus osteoglycin (Ogn) | NM_008760 | 0.361* |
| Mus musculus lumican (Lum) | NM_008524 | 0.436* |
| Mus musculus elongation of very long chain fatty acids (FEN1/Elo2, SUR4/Elo3, yeast)-like 2 | NM_019423 | 2.100* |

| | | |
|---|---|---|
| (Elovl2) | | |
| Mus musculus CD84 antigen (Cd84), transcript variant 2 | NM_001252472 | 0.448* |
| Mus musculus C-type lectin domain family 4, member d (Clec4d), transcript variant 2 | NM_001163161 | 2.031* |
| Unknown (93) | | |
| Data not shown | | |
| The log ratio is defined as log2 (EdTx-treated group / PBS-treated group) | | |

*$p < 0.05$ and **$p < 0.01$ versus PBS-treated group.
