# Supplementary material for: Identification of Potential Therapeutic Targets Against Anthrax-Toxin-Induced Liver and Heart Damage
Source: Toxins (Basel). 2025 Jan 24;17(2):54. doi: 10.3390/toxins17020054 (PMC11861023; doi:10.3390/toxins17020054)
Supplement: Supplementary file 1 [file toxins-17-00054-s001.zip › Supplementary Table S3.pdf]

Supplementary Table S3. Efficiency of gene knockdown in mouse primary hepatocytes after EdTx challenge

	CMG2	Ramp3	Rgs1	Pck1	G6pc	Hcar2	Fosl2	Fos	Cxcl2	Cxcl3
si-no	0.00	0.00	0.00	0.00	0.00	0.00	0.00	0.00	0.00	0.00
si-GFP	-0.10	-4.49	4.52	0.56	9.93	-0.01	2.84	-2.91	3.94	8.41
si-CMG2	75.47	-	-	-	-	-	-	-	-	-
si-Ramp3	-	43.99	-	-	-	-	-	-	-	-
si-Rgs1	-	-	68.11	-	-	-	-	-	-	-
si-Pck1	-	-	-	38.32	-	-	-	-	-	-
si-G6pc	-	-	-	-	37.08	-	-	-	-	-
si-4mix	-	41.53	62.55	43.15	38.16	-	-	-	-	-
si-Hcar2	-	-	-	-	-	50.22	-	-	-	-
si-Fosl2	-	-	-	-	-	-	45.65	-	-	-
si-Fos	-	-	-	-	-	-	-	43.38	-	-
si-cCxcl2	-	-	-	-	-	-	-	-	81.57	-

si-cCxcl3	-	-	-	-	-	-	-	-	-	91.35
si-5mix	-	-	-	-	-	39.75	36.48	56.72	65.19	87.30

Note: Representative results from three independent experiments are shown.
