# Supplementary material for: Identification of Potential Therapeutic Targets Against Anthrax-Toxin-Induced Liver and Heart Damage
Source: Toxins (Basel). 2025 Jan 24;17(2):54. doi: 10.3390/toxins17020054 (PMC11861023; doi:10.3390/toxins17020054)
Supplement: Supplementary file 1 [file toxins-17-00054-s001.zip › Supplementary Table S4.pdf]

Supplementary Table S4. Sequence information of siRNA used in this study

Name	Catalog no.	Duplexe	Sequences	
RAMP3 siRNA(m)	SC-40897	A	Sense	GGUUCAGAUUGUCCAUACUtt
			Antisense	AGUAUGGACAAUCUGAACCtt
		B	Sense	CUGAGCACAUCAUUUAUCAtt
			Antisense	UGAUAAAUGAUGUGCUCAGtt
		C	Sense	CUCUGUGAUCUGUCACGAUtt
			Antisense	AUCGUGACAGAUCACAGAGtt
RGS1 siRNA(m)	SC-36409	A	Sense	GACUCAGAAAGGAUUAACAtt
			Antisense	UGUUAAUCCUUUCUGAGUCtt
		B	Sense	GGAAGCAUGAACAAGAAGAtt
			Antisense	UCUUCUUGUUCAUGCUUCCtt
		C	Sense	GAAGCAUGAACAAGAAGAAtt
			Antisense	UUCUUCUUGUUCAUGCUUCtt
PCK1 siRNA(m)	SC-76107	A	Sense	CCAUGUAUGUCAUCCCAUUtt
			Antisense	AAUGGGAUGACAUACAUGGtt
		B	Sense	GCAACUUAAGGGCUAUCAAtt
			Antisense	UUGAUAGCCCUUAAGUUGCtt
		C	Sense	CAGAAGGAGUACCCAUUGAtt
			Antisense	UCAAUGGGUACUCCUUCUGtt
G6PC siRNA(m)	SC-145294	A	Sense	CAUGCAGAGUCUUUGGUAUtt
			Antisense	AUACCAAAGACUCUGCAUGtt

		B	Sense	GCAAACCAGAUGCAAUCUAtt
			Antisense	UAGAUUGCAUCUGGUUUGCtt
		C	Sense	CUCUAUCACGUCACAGUUUtt
			Antisense	AAACUGUGACGUGAUAGAGtt
HCAR2 siRNA(m)	SC-60793	A	Sense	CACACCACUUCUUGAACAAtt
			Antisense	UUGUUCAAGAAGUGGUGUGtt
		B	Sense	CUAUGUUCCUCUUGGAAUUtt
			Antisense	AAUUCCAAGAGGAACAUAGtt
		C	Sense	CUGUCUGCGUUUCUUAGUAtt
			Antisense	UACUAAGAAACGCAGACAGtt
FOSL2 siRNA(m)	SC-35408	A	Sense	GUCUCUUCUUGCUUCUAGUtt
			Antisense	ACUAGAAGCAAGAAGAGACtt
		B	Sense	CACCAAAUGUCUGUAAUGAtt
			Antisense	UCAUUACAGACAUUUGGUGtt
		C	Sense	CUAGGUAUCAGAUUCCUUUtt
			Antisense	AAAGGAAUCUGAUACCUAGtt
FOS siRNA(m)	SC-29222	A	Sense	GGUAGUUAGUAGAGCAUGUtt
			Antisense	ACAUGCUCUACUAACUACCtt
		B	Sense	CUCCUGAAGAGGAAGAGAAtt
			Antisense	UUCUCUUCCUCUUCAGGAGtt
		C	Sense	CGGAGACAGAUCAACUUGAtt
			Antisense	UCAAGUUGAUCUGUCUCCGtt

		D	Sense	CACCUCUUCCAGAGAUGUAtt
			Antisense	UACAUCUCUGGAAGAGGUGtt
CXCL2 siRNA(m)	SC-45997	A	Sense	CAAGGGUUGACUUCAAGAAtt
			Antisense	UUCUUGAAGUCAACCCUUGtt
		B	Sense	GAUGCUGGAUUUCAAUGUAtt
			Antisense	UACAUUGAAAUCCAGCAUCtt
CXCL3 siRNA(m)	SC-142642	A	Sense	GGGUAUAAUUGCAUCUACUtt
			Antisense	AGUAGAUGCAAUUAUACCCtt
		B	Sense	GCAUGUGCACAUCUAGUUUtt
			Antisense	AAACUAGAUGUGCACAUGCtt
CMG2 siRNA (m)	Sc-60232	A	Sense	GUGUGACAGUGUAUCUUCAtt
			Antisense	UGAAGAUACACUGUCACACtt
		B	Sense	CGACAUGAGAGGUGAUGAAtt
			Antisense	UUCAUCACCUCUCAUGUCGtt
		C	Sense	GAAGGAAAUAGCUCAGAUAtt
			Antisense	UAUCUGAGCUAUUUCCUUCtt
