# Supplementary material for: Identification of Potential Therapeutic Targets Against Anthrax-Toxin-Induced Liver and Heart Damage
Source: Toxins (Basel). 2025 Jan 24;17(2):54. doi: 10.3390/toxins17020054 (PMC11861023; doi:10.3390/toxins17020054)
Supplement: Supplementary file 1 [file toxins-17-00054-s001.zip › Supplementary Table S5.pdf]

Supplementary Table S5. Primers used for qPCR in this study

	Gene bank ID	Primers (5'-3')	
Arg2	NM_009705	sense	CCCACAAGATGATCCCTACAAT
		antisense	CACCTGACACAGCTCTACTAAC
Ass1	NM_007494	sense	GAAGAGCTGGTGAGCATGAA
		antisense	AGCCTGAGCGAGTTGATATTG
Cbs	NM_001271353	sense	TCCTAACACCCAGCTACCTAAA
		antisense	CCATCCTTCCTGGCTAACATTC
Cps1	NM_001080809	sense	GAGACGAACTGGGACTGAATAA
		antisense	GTAGCCAGCCAGTGGTTATAG
Got1	NM_010324	sense	CCCAAGCAGGTCGAGTATTT
		antisense	TGGAGGTAGCGACGTAATCTA
Pah	NM_008777	sense	ATACACAGAGGAGGAGAGGAAG
		antisense	AAGAGGGAAGATGTGGTTGTG
Tat	NM_146214	sense	CCTGGACAGAACATCCTCATTC
		antisense	CCCAAGACTTCTCAGGCAATAG
Gfpt1	NM_013528	sense	GCAAGAGAGACGCAAAGAGA
		antisense	GACCGACTTCTGGTGGTAAAG
Gfpt2	NM_013529	sense	GTCCTCCGAGGTTATGATGTTG
		antisense	GGTGACGACAGTCTTGTGATAG
Akr1b7	NM_009731	sense	CCTGAACAAGCCTGGACTAAA

		antisense	GATGCCCTTGGATTGACAGTA
B3gnt5	NM_001159407	sense	CTGCACTACCCATCCATTGT
		antisense	TTTCCCACAGTCACAGCATAG
Crem	NM_001110850	sense	GAAGAAGGGACACCACCTAAC
		antisense	GTCACCTGTGGCAGTGTATT
Csf2rb	NM_007780	sense	GGTCAAGCCCATCTCTAACTAC
		antisense	GGATGAGAAAGACCAGGATGAG
Csf2rb2	NM_001287389	sense	AGCTCTGCATGGTCTGTTTAG
		antisense	GGCAGAAATGTGCTGTGTTATC
Cyp17a1	NM_007809	sense	ACCAGCCAGATCGGTTTATG
		antisense	TAACTGGGTGTGGGTGTAATG
Dgat1	NM_010046	sense	GGCCTTACTGGTTGAGTCTATC
		antisense	GTTGACATCCCGGTAGGAATAA
Entpd1	NM_009848	sense	GCCCTAACTCAAGCTGTCTATC
		antisense	GATTCAGGACACTTGGCTTCTA
Etnppl	NM_001163587	sense	GGGACTTTGATTCTGGCTACTC
		antisense	CAGTGGGATGCAGGTGATAAT
G6pc	NM_008061	sense	GCATTTGCCAGGAAGAGAAAG
		antisense	AACTGAAGCCGGTTAGACATAG
Gem	NM_010276	sense	TGTGAGGTCTTGGGAGAAGATA
		antisense	CCACATGTCCAGGAGGATAATG

Hpgds	NM_019455	sense	GCTCCTAGTGTTGCTGTGAA
		antisense	CCCTGTAAGCTGTTGTGTATCT
Lepr	NM_001122899	sense	GAGATGGCTCAGTGGTTAAGAG
		antisense	GGATTTCATTACGGATGGTTGTG
Pck1	NM_011044	sense	TTTGTAGGAGCAGCCATGAG
		antisense	CCGAAGTTGTAGCCGAAGAA
Pde3b	NM_011055	sense	GTCTGCTGGCTCTCTAACTAATC
		antisense	GAAATCTGCTGCACTTGATACAC
Pde4b	NM_001177980	sense	GGAGAAGGCCACAGCTATTT
		antisense	CCACACAGAGGGAGAGAGATTA
Ppargc1a	NM_008904	sense	GACAATCCCGAAGACACTACAG
		antisense	AGAGAGGAGAGAGAGAGAGAGA
Ptprn	NM_008985	sense	ATCTTCCCTCTACCACGTCTAT
		antisense	GGTCTGCAGGTTCTTAAGGTAG
Ramp3	NM_019511	sense	TTGGGCTAGTGGAAGAAAGTG
		antisense	CTGCCAAGAAACGGCTAGAA
Rdh12	NM_030017	sense	GGATCCTGGGAAGTTGGATTAG
		antisense	CTAGAGCTGGAGGGAATAGAAATG
Rgs1	NM_015811	sense	CGAGAATCGACAGCCAAGAA
		antisense	TGATTTCAGGAACCTGGGATAAG
Rgs2	NM_009061	sense	TCGGGAAAGCAGAGTTTGAG

		antisense	CCAACTAGCTAAGGCCACATAA
Sgk1	NM_001161845	sense	CTAGGCACAAGGCAGAAGAA
		antisense	AGAACATTCCGCTCTGACATAA
Sik1	NM_010831	sense	AAACCTCCCTTGGTCACATTAG
		antisense	TCTTCCCTGACACCTCTACTC
Slc25a25	NM_001164357	sense	GGACCGGGAGGATTTCTTTATT
		antisense	CTTCAGTCCTCACCCTCAAAC
Tbxas1	NM_011539	sense	TTCACATACCTGCCCTTTGG
		antisense	CTTGTGTAGGACCTGGAGTATTG
Tgfbr1	NM_009370	sense	CCTTGAGTCACTGGGTGTTATG
		antisense	CCACTTAGCTGTCACCCTAATC
Tgm2	NM_009373	sense	CATCACCAGCACTCTGTATCTC
		antisense	GGTTCCTTCGGTTCCTTCAT
Uap1	NM_133806	sense	GAGAGGGCCTTGAAGGTTATG
		antisense	CTATGTGGCCCGTTAGGATTT
Uck2	NM_030724	sense	CGAGACCTGTTCCAGATGAAG
		antisense	TTCGCTGATGTCCCTCAATAC
BMP7	NM_007557	sense	AGAGGTGGGATGTTGGTTATG
		antisense	CCAGTTTAACCCTCTGCATTTG
C3ar1	NM_009779	sense	GAGCAAGTGAGCACAGATACA
		antisense	GCAGAATACACAGGGAAGAGAG

Cd180	NM_008533	sense	CTCCGAAACCTGTCTCACTTAC
		antisense	GTTCTAGCTGAGGGCATTCTT
Cd55	NM_010016	sense	GACAGACAGACAGACAGACATAC
		antisense	GTCTCCAACCACTTCCTCTTAAT
Cd86	NM_019388	sense	CCTGGAAAGGTCTGGAGAATG
		antisense	GGCAGATCAGTCCTTCCATAAA
Cxcl2	NM_009140	sense	TAAGCACCGAGGAGAGTAGAA
		antisense	GTCCAAGGGTTACTCACAACA
Cxcl3	NM_203320	sense	GCACCCAGACAGAAGTCATAG
		antisense	ACTTGCCGCTCTTCAGTATC
Cyr61	NM_010516	sense	CCAGTGTACAGCAGCCTAAA
		antisense	CTGGAGCATCCTGCATAAGTAA
Egln3	NM_028133	sense	GCCCAGGACTGCTTCTTATT
		antisense	TGGCATCTGTCACCAACTTTA
F5	NM_007976	sense	GTCCAGTTTATCCTCTGCTCTTG
		antisense	CACAGGTCACAGTCCCTTATTG
Fos	NM_010234	sense	GAATCCGAAGGGAACGGAATAA
		antisense	TCTCCGCTTGGAGTGTATCT
Fosl2	NM_008037	sense	GCCTGCTTGCTTTGTCTTAC
		antisense	GAGGTCACACCCAGAGTTTAG
Hcar2	NM_030701	sense	CCTTATCTGGCTTCCACATCTC

		antisense	GTTCAACGAACGGCCAAATC
Hilpda	NM_001190461	sense	GCAGGATCTAGCAGCAGAAA
		antisense	CATGATGCCCAGCACATAGA
Igf1	NM_001111274	sense	GCTGCTGAAGCCATTCATTTAG
		antisense	CGTGGGAAGAGGTGAAGATAAG
Il11	NM_008350	sense	GGGATCACCTGTGGCTTATT
		antisense	GATCTCAGTTCCCTGCTCTTC
Il1b	NM_008361	sense	ATGGGCAACCACTTACCTATTT
		antisense	GTTCTAGAGAGTGCTGCCTAATG
Il1r2	NM_010555	sense	CTGATAGTCCCGTGCAAAGT
		antisense	GGGTAAGCAGCCGAGATAAA
Il33	NM_001164724	sense	CCTACTCCCTCAGCTTTCTTTC
		antisense	GCAGGGTAAAGACAGTGGAATA
Il6	NM_031168	sense	GTCTGTAGCTCATTCTGCTCTG
		antisense	GAAGGCAACTGGATGGAAGT
Il7r	NM_008372	sense	GCGTATGTCACCATGTCTAGTT
		antisense	AGCATTCCAGACTTTCCATCTC
Ngf	NM_001112698	sense	CAGTGAGGTGCATAGCGTAAT
		antisense	CTCCTTCTGGGACATTGCTATC
Nr4a2	NM_001139509	sense	CAGAGCTACAGTTACCACTCTTC
		antisense	TGGTGAGGTCCATGCTAAAC

Nr4a3	NM_015743	sense	CTCAGTGTCGGGATGGTTAAG
		antisense	CCTGTTGTAGTGGGCTCTTT
Procr	NM_011171	sense	GCCTCCCTTCTCTTTCCTAATC
		antisense	GGCAGAAACTTCGTCAACATC
Rasgef1b	NM_145839	sense	GAGCGAGGATGATCGAGTATTT
		antisense	CGGGCTCATATTCATACCAGAG
Star	NM_011485	sense	GCTGTGAAGGCTAAGGGATAAG
		antisense	GTGACATTTGGAGCTGGTAAGA
Tlr7	NM_133211	sense	GCCATCCAGCTTACATCTTCT
		antisense	TTTGACCCAGGTAGAGTGTTTC
Tnfaip6	NM_009398	sense	TGGCCTCGAACTCAGAAATC
		antisense	CGAGGTCCAAGAGCTACAAATA
Trem1	NM_021406	sense	GTCCAGTTTATCCTCTGCTCTTG
		antisense	CACAGGTCACAGTCCCTTATTG
Vegfa	NM_001025250	sense	TGGTTCTTCACTCCCTCAAATC
		antisense	GGTCTCTCTCTCTCTTCCTTGA
Ucp3	NM_009464	sense	CATCAGGGTGTTGGGAAGATAG
		antisense	CATTGTCCTCAGGCTTACATTTG
Egln3	NM_028133	sense	GCCCAGGACTGCTTCTTATT
		antisense	TGGCATCTGTCACCAACTTTA
Gp49a	NM_008147	sense	CTGTCAGTCTATCCCAGCTCTA

		antisense	CCATGCTTTCCTTCCTGTATCA
Map2k6	NM_011943	sense	GGATACGGGCCACAGTTAATAG
		antisense	GTAGAAGGTCACGGTGAATGG
Mmp12	NM_008605	sense	GACATCTTGGCTCCCTATCTTC
		antisense	TGGACAATACACCAGTCAGTTT
Rcan1	NM_001081549	sense	CCGACAAACAGTTCCTCATCT
		antisense	CCAGCTTGGAGATGGCATATAA
Hbegf	NM_010415	sense	CTGGGTCCTATTTGCTCTGTAA
		antisense	CTCTGACCATACACAACCTACC
Sprr1a	NM_009264	sense	CTGAAGACCTGATCACCAGATG
		antisense	GTGCAAGGAGAGAGGGATTAAG
Lilrb4	NM_013532	sense	CCCTCTGGAAACCAGGAATAAG
		antisense	CCAGCAGCACTCTCATAGTAAC
Bmp10	NM_009756	sense	CTGGGTATGAAGCCTATGAGTG
		antisense	GTGGACCAAGGCCTGAATAA
Abra	NM_175456	sense	CTGTAAGGCCCATCGGAAATA
		antisense	ACTGAAGGGATTGAGCTTCTG
Ctgf	NM_010217	sense	CAAATGCTGTGCAGGTGATAAA
		antisense	CCTGAGCCAGCCATTTCTTA
Nppb	NM_001287348	sense	ACCACCTTTGAAGTGATCCTATT
		antisense	GCAAGTTTGTGCTCCAAGATAAG

Dusp1	NM_013642	sense	CATGGGAGCTGGTCCTTATTT
		antisense	CTTGCGGTCAAGTCATTGTTG
Ier3	NM_133662	sense	GGTCACAGTCCGAAGAAACA
		antisense	CTGAGTTAGCGTTGCCTTAGA
Serpine1	NM_008871	sense	GGGACGAAACTGGAGATGTTAT
		antisense	GAGGAGTTGCCTTCTCTTTCTC
Ptgs2	NM_011198	sense	CGGACTGGATTCTATGGTGAAA
		antisense	CTTGAAGTGGGTCAGGATGTAG
Tnfrsf12a	NM_001161746	sense	CATAGAGGAGACTGGTGGAGA
		antisense	AGGCTGACTCCAGAATGAATG
GAPDH	NM_001289726	sense	AACAGCAACTCCCACTCTTC
		antisense	CCTGTTGCTGTAGCCGTATT
GAPDH (PCR only)	Arevalo et al., 2014 [15]	sense	TGAAGGTCGGTGTGAACGGATTTGGC
		antisense	TAGTGGGGTCTCGCTCCTGGAAGATG
Cmg2	Arevalo et al., 2014 [15]	sense	CTGACAGAGAGATTTGTGAGC
		antisense	GCAATTCTTTCCAGCTGA
