# Supplementary material for: Identification of Potential Therapeutic Targets Against Anthrax-Toxin-Induced Liver and Heart Damage
Source: Toxins (Basel). 2025 Jan 24;17(2):54. doi: 10.3390/toxins17020054 (PMC11861023; doi:10.3390/toxins17020054)
Supplement: Supplementary file 1 [file toxins-17-00054-s001.zip › Supplementary Table S6.pdf]

Supplementary Table S6. LeTx-mediated effects on the expression of selected genes in mouse primary cardiomyocytes, HL-1 cells, primary hepatocytes, mouse livers and mouse heart. The expression changes (log ratio) in comparison to the untreated controls are given.

**Gene name**	**Genebank ID**	**Mouse primary cardiomyocytes**		**HL-1**	**Mouse heart**	**Primary hepatocytes**	**Mouse liver**
		**Microarray**	**qPCR**	**qPCR**	**qPCR**	**qPCR**	**qPCR**
Mus musculus actin-binding Rho activating protein (Abra), mRNA	NM_175456	0.422^{*}	0.282^{*}	0.776	0.678^{*}	1.251	1.003
Mus musculus bone morphogenetic protein 10 (Bmp10), mRNA	NM_009756	0.337^{**}	0.278^{**}	0.847	0.280^{**}	1.408	0.421^{**}
Mus musculus connective tissue growth factor (Ctgf), mRNA	NM_010217	0.489^{*}	0.339^{**}	0.858	0.378^{**}	0.467^{*}	0.763
Mus musculus dual specificity phosphatase 1 (Dusp1), mRNA	NM_013642	0.477^{*}	0.392^{*}	0.461^{*}	0.496^{**}	0.481^{*}	3.282^{**}
Mus musculus egl-9 family hypoxia-inducible factor 3 (Egln3), mRNA	NM_028133	0.435^{*}	0.500^{**}	0.487^{*}	0.568^{**}	1.528	1.451
Mus musculus glycoprotein 49 A (Gp49a), transcript variant 1, mRNA	NM_008147	0.523^{*}	0.367^{*}	0.912	0.371^{**}	0.145^{**}	0.425^{*}
Mus musculus heparin-binding EGF-like growth factor (Hbegf), mRNA	NM_010415	0.440^{*}	0.404^{*}	0.433^{**}	0.497^{*}	0.476^{*}	0.410^{**}
Mus musculus immediate early response 3 (Ier3), mRNA	NM_133662	0.450^{*}	0.409^{*}	1.395	0.480^{*}	0.499^{**}	7.247^{**}

Mus musculus leukocyte immunoglobulin-like receptor, subfamily B, member 4 (Lilrb4), transcript variant 1, mRNA	NM_013532	0.481*	0.469*	0.879	0.186**	0.046*	0.201*
Mus musculus mitogen-activated protein kinase kinase 6 (Map2k6), mRNA	NM_011943	2.835**	5.554**	2.291*	4.069**	2.174*	4.121**
Mus musculus matrix metallopeptidase 12 (Mmp12), mRNA	NM_008605	0.266**	0.414*	1.015	1.533	2.155	1.334
Mus musculus natriuretic peptide type B (Nppb), transcript variant 2, mRNA	NM_001287348	0.377	0.240**	0.020**	0.008**	0.233*	1.179
Mus musculus prostaglandin-endoperoxide synthase 2 (Ptgs2), mRNA	NM_011198	0.485**	0.301*	0.884	0.647	0.996	0.937
Mus musculus regulator of calcineurin 1 (Rcan1), transcript variant 1, mRNA	NM_001081549	0.485**	0.350**	0.430**	0.425**	1.252	0.298**
Mus musculus serine (or cysteine) peptidase inhibitor, clade E, member 1 (Serpine1), mRNA	NM_008871	0.483*	0.423*	1.019	0.389*	0.580	327.778**
Mus musculus small proline-rich protein 1A (Sprr1a), mRNA	NM_009264	0.428**	0.331*	0.445**	0.597	0.132**	0.344*
Mus musculus tumor necrosis factor receptor superfamily, member 12a	NM_001161746	0.437	0.480*	0.162**	0.119**	0.401**	0.397*

(Tnfrsf12a), transcript variant 2, mRNA							
Mus musculus uncoupling protein 3 (mitochondrial, proton carrier) (Ucp3), mRNA	NM_009464	1.911	3.723*	1.016	4.636*	2.891	0.518
Mus musculus a disintegrin-like and metallopeptidase (reprolysin type) with thrombospondin type 1 motif, 8 (Adamts8), mRNA	NM_013906	0.545*					
Mus musculus predicted gene 12409 (Gm12409), long non-coding RNA	NR_046068	1.970*					
Mus musculus keratin 18 (Krt18), mRNA	NM_010664	0.552**					
Mus musculus mesoderm specific transcript (Mest), transcript variant 1, mRNA	NM_001252292	0.520**					
Mus musculus microRNA 181b-2 (Mir181b-2), microRNA	NR_029904	1.871*					
Mus musculus serine (or cysteine) peptidase inhibitor, clade B, member 1a (Serpinb1a)	NM_025429	0.532*					
Mus musculus solute carrier family 38, member 2 (Slc38a2), mRNA	NM_175121	0.539*					

Mus musculus signal peptidase complex subunit 3 homolog (S. cerevisiae) (Spcs3), mRNA	NM_029701	0.535*					
Unknown (26) not show							

*$p < 0.05$ and **$p < 0.01$ versus the PBS-treated group.
